# Social isolation, social exclusion, and access to mental and tangible resources: mapping the gendered impact of tuberculosis-related stigma among men and women living with tuberculosis in Eastern Cape Province, South Africa

**DOI:** 10.1186/s44263-025-00166-6

**Published:** 2025-06-05

**Authors:** Andrew Medina-Marino, Lindsey de Vos, Joseph Daniels

**Affiliations:** 1 Desmond Tutu HIV Centre, University of Cape Town, 3 Woodlands Road, Woodstock, Cape Town, 7925 South Africa; 2 Department of Psychiatry, Perelman School of Medicine, University of Pennsylvania, Philadelphia, USA; 3 Edson College of Nursing and Health Innovation, Arizona State University, Phoenix, AZ USA

**Keywords:** Tuberculosis, Stigma, Gender, Qualitative, South Africa

## Abstract

**Background:**

In 2022, an estimated 10.6 million people developed tuberculosis (TB) globally, with men bearing a greater burden of disease compared to women. In South Africa specifically, men experience higher risks of poor outcomes and TB-related mortality than women. Stigma and isolation among people living with tuberculosis (PLWTB) are well documented. The gendered pathways through which TB-related stigma leads to isolation or impacts access to resources during one’s illness-to-health journey are poorly understood.

**Methods:**

We interviewed PLWTB receiving treatment at government clinics in Buffalo City Metro Health District, Eastern Cape Province, South Africa. Semi-structured guides explored TB symptom experiences, access to care, treatment motivation, key supporters, and access to mental and tangible resources (MTRs) during illness. Open coding was done inductively, with MTR domains informed by the Network-Individual-Resource Model. Findings were analyzed through a cyclic, iterative, and deductive process using social isolation and exclusion as interpretive lenses. Memos and pathway mapping examined gendered differences in stigma, isolation, and access to networked MTRs.

**Results:**

One hundred forty-two PLWTB (men = 86; women = 56) were interviewed. PLWTB described pervasive TB stigma and isolation. Women described self-isolating in response to enacted and anticipated stigma. Men described active exclusion by friends and family. Women’s maintenance of familial ties facilitated access to MTRs while ill. Men’s systematic exclusion (e.g., deliberate or forced by peers or family) reduced their agency to access resources. Men and women described regaining physical strength and recovery of social networks through treatment, but also the sustained impacts of post-treatment stigma.

**Conclusions:**

We identified gendered pathways through which TB stigma and isolation affect access to MTRs. For women, stigma led to social isolation, but familial networks helped maintain access to MTRs, fostering resilience. Men experienced social exclusion, reduced agency to access MTRs, and increased vulnerability during illness. Findings can guide gender-responsive interventions to reduce the impact of TB stigma on health outcomes.

**Supplementary Information:**

The online version contains supplementary material available at 10.1186/s44263-025-00166-6.

## Background

Globally, an estimated 10.6 million people developed TB in 2022, of those, 55% were adult men, 33% were adult women, and 12% were children [1]. South Africa is one of 30 high-burden countries, accounting for 87% of global TB incidence. Additionally, it is classified as a high-burden country for HIV co-infection, and rifampicin-/multi-drug-resistant TB [1]. Compared to women worldwide, men are at increased risk for poor outcomes along the TB care cascade including TB incidence, delayed healthcare seeking, and TB-related mortality [1–4]. Though women bear a lower burden of TB disease, TB exacerbates the gendered health inequalities and vulnerability that women face globally, which increases their risk of poor TB outcomes [5–7]. A host of social determinants, including stigma, gender norms and practices, social support, and access to tangible (e.g., food, money, transportation) and mental (i.e., self-efficacy, coping mechanisms, resiliency) resources are known to influence both men and women’s risk for poor treatment outcomes [8–12]. However, elucidating gender-specific dimensions in how men and women experience TB illness, particularly TB-related stigma and isolation, is necessary to inform gender-responsive interventions to improve men and women’s TB-related health outcomes and quality of life during their illness-to-health journey [9, 10, 13, 14].

Stigmatization of people with TB are driven by a combination of misconceptions, cultural beliefs, social dynamics and judgement, and fear of contagion, all of which results in distinctive acts of exclusion beyond advised TB protocols [15–20]. Perceived and real signs and symptoms of TB, especially cough and weight loss, may thus bring about prolonged isolation stemming from self-imposed or enforced isolation by others [17, 19]. Such acts include self-withdrawal due to perceived social repercussions and community stigma [17, 20], not sharing utensils with others and being excluded from social and familial events and networks, all of which can impact a patient’s quality of life and resiliency [7, 17, 21–23]. Although there are similarities in how men and women experience TB-related stigma, few studies report higher TB stigma or vulnerability among men [14, 24]. Much more often, women are reported to experience higher stigma burden due to gender and cultural norms that increase women’s vulnerability and social inequities, especially in highly patriarchal societies that further impose barriers to accessing health services [5, 15, 22, 25, 26]. Stigmatizing perceptions can further impact men and women’s health-care seeking behavior, retention in care and preferences for TB care and treatment delivery [14, 21, 25, 27–32].

The ability of individuals living with TB to navigate stigma during their illness-to-health journey is influenced by psychosocial support and access to resources [9, 33–35]. Support networks, including partners, family, and community members, offer essential provisions like emotional support, food for treatment intake and means to access healthcare such as money or transportation [23, 34, 36–38]. Similarly, supportive healthcare providers who provide sufficient information and regular treatment pick-up reminders encourage individuals to seek care. Alternatively, unsupportive networks can restrict or block access to resources through isolation and exclusion. This may result in reduced resiliency to cope with TB illness [33], an inability to practice optimal health behaviors, and negatively impact the quality of life of those living with TB, all of which have a direct impact on treatment adherence and completion [12, 19, 20, 37, 39].

A complex interplay between stigma and gender, and their intersecting impact on resource access and support, have been described for other health conditions [16, 40–42]. Given that men and women’s TB illness and treatment experiences may vary greatly due to social and cultural variations, including existing gender norms [43–45], we explored differences by gender, how men and women living with TB in South Africa experience stigma and isolation, and how these impact their social networks and access to resources during treatment. An isiXhosa translation of the abstract is provided in Additional file 1.

## Methods

This qualitative substudy was nested within a larger, mixed-methods, prospective cohort study (NIH R21 AI148852; https://reporter.nih.gov/project-details/10085625), which sought to identify gender-specific and gender-neutral preferences for a male-centered TB care and support intervention. The report of the current study follows the consolidated criteria for reporting qualitative studies (COREQ) guideline (Additional file 2) [46].

### Study framework

This study was guided by the Network-Individual-Resource Model (NIRM) and concepts of isolation and stigma [47–50]. NIRM was used to examine the complex interplay between individual- and network-level resources that shape health-related behaviors. Its ecological approach and theoretically based framework offers a robust lens through which to explore and understand men’s and women’s engagement (or lack thereof) in TB care and to inform the development of contextually relevant, resource-sensitive interventions [47, 51]. The NIRM conceptualizes resources into mental (e.g., personal agency, attitudes, perceived norms, social support, social capital) and tangible (e.g., income, access to food, physical health, transport), operationalized at the individual and network levels [47]. The NIRM postulates that networks and network membership can mitigate risk and enhance coping mechanisms by providing relevant resources, but can be strained by stigma during illness. When stigma leads to social isolation, access to resources is disrupted, weakening coping capacity and increasing vulnerability to poor outcomes [33]. Within this framework, agency refers to an individual’s ability to access resources or maintain control over their actions [52], while resiliency denotes the capacity to engage or mobilize networks despite adversity [53]. The NIRM emphasizes that a lack of tangible resources can gradually deplete mental well-being, whereas continued access sustains one’s mental well-being [47, 54, 55]. Stigma can compound agency, destabilize networks, and manifest as forms of isolation [16].

Isolation among those affected by TB is well-reported in TB literature; however, we sought to differentiate the concepts of self-imposed isolation and social exclusion [17–19, 37, 56], related but distinct experiences in the realm of social relationships. Social isolation refers to the objective state of limited social contact which can be voluntary (i.e., retreating from social interactions to minimize stigmatization or to minimize infecting others) or involuntary (i.e., weakness due to illness; geographic remoteness) [48, 49]. In comparison, social exclusion stems from societal structures, discrimination, or prejudice and refers to the marginalization of individuals or groups including restricted access to resources, opportunities, or social participation [48, 49]. As a result, social exclusion can impact one’s resiliency, including coping, and manifest as internalized stigma (reduced mental well-being). These concepts were used as interpretive lenses to explore how different forms of isolation influence resource access and vulnerability during TB illness [48, 49]. In this context, we used the NIRM to explore and describe pathways by which isolation influences access to resources and, to better integrate stigma into the model, drew on existing literature to define stigma types (e.g., anticipated, enacted, and internalized) [50, 57–59].

### Study setting

This study was conducted in Buffalo City Metropolitan (BCM) Health District, Eastern Cape Province, South Africa. Eastern Cape Province is historically under-resourced and lacks the mature research infrastructure of historically advantaged areas of Durban, Johannesburg, and Cape Town. Since 1990, Eastern Cape has ranked last among South Africa’s nine provinces in its Human Development Index score [60, 61]. In South Africa, TB is linked to poverty, poor living conditions, and limited healthcare access, where the impact of illness on income loss and resource depletion further exacerbate financial strain [62–64]. Further, those living with TB have to pay for their own transport and nutritional support. Patients unable to work for 6 months may receive a disability grant (often based on clinical evaluations and limited by structural barriers), while other temporary options include the Social Relief of Distress grant. Those affected by TB often report the need for food parcels and are sporadically offered by civil society organizations and food (nutrition) programs [12]. Given limited external support or social protection many depend on family for support and essential resources during treatment, especially when unable to work or requiring increased nutritional food for treatment intake. Lack of food or support can result in missed treatment and poorer health [12, 62]. In 2019, the estimated HIV prevalence in Eastern Cape Province was 20.2% (95% CI 19.7–20.9%) among those aged 15–49 years [65, 66]. That same year, BCM had an estimated TB incidence of 876 per 100,000 population (male incidence = ~ 1072 per 100,000 population; female incidence = ~ 540 per 100,000 population) [67]. In 2018, the last year in which data were published, BCM had a drug-susceptible TB (DS-TB) treatment success rate of 71.2% (the lowest in South Africa), a loss-to-care (LTC) rate of 17.6% (second highest in South Africa), and a TB client death rate of 6.3% [68].

### Participant recruitment

From March 2021 through January 2022, study staff were embedded in 15 of 79 primary and community health clinics throughout BCM, including (1) urban, peri-urban, and rural clinics and (2) clinics with catchment areas including formal and informal settlement housing. In collaboration with clinic TB nurses, individuals on TB treatment were identified and screened for eligibility by study staff. Inclusion criteria included (1) age ≥ 18 years, (2) currently living in BCM, and (3) currently engaged in or initiating treatment for DS-TB. Exclusion included (1) 17 years or younger and (2) any extra-pulmonary TB without lung involvement. All eligible individuals (adult men and women) were invited to learn about the study, consecutively recruited, consented and administered a study questionnaire by study staff at time-of-enrollment. Participants were prospectively followed and purposively invited for an in-depth interview (IDI) based on their progression along the TB treatment cascade (i.e., currently on treatment; missed ≥ 15 days of a treatment refill visit; recently completed treatment). The study attempted to recruit an equal number of men and women on TB treatment. Given South Africa’s high TB burden, the study aimed to recruit up to 30 participants of each gender at each stage of the TB care cascade to ensure a sufficiently representative sample stratified by gender and treatment experience. This approach also sought to better understand gender-centered TB care preferences. Participants were those successfully contacted by interviewers and willing to participate in an IDI.

### Data collection

Semi-structured interview protocols were developed to examine the following domains: experiences and perspectives of TB symptoms, access to clinical care and services, individual motivations for treatment, disclosure decision-making, social and familial network support during TB illness, perspectives of other men and women’s TB experiences, and probing for tangible and mental resources accessed during their TB illness and treatment journey. Open-ended questions in line with these domains further explored changes in the behaviors and dynamics of their social and familial networks, experiences with anticipated, enacted and internalized stigma, judgment, the ability to access support, perceived impacts of their illness on their lifestyle and relationships, and experiences of isolation and network membership exclusion (Additional file 3).

Interviews were scheduled with participants either telephonically or via home visits. Interviews were conducted in a participant’s preferred language (English or IsiXhosa) and in a private location agreeable to the study participant (i.e., within the comfort of their own home). Interviews were audio-recorded and lasted approximately 60–90 min. The gender of the interviewers was matched to that of the participants. Interviewers were South African native isiXhosa speakers with public health or social work experience, and knowledge of the health district that enabled them to engage participants in a culturally sensitive manner. Interviewers received good clinical practice training from management staff before study implementation, and interviewing skills training from qualitative research experts before conducting the in-depth interviews. Interviewers had no prior relationships with participants. Data collection was monitored and refined through a review of initial interviews and weekly team meetings. Refresher trainings provided interviewers with an opportunity to share real-time experiences, as well as offer supervision and initial feedback on the interview guides. Recording of interviews was voluntary. Interview participants were provided a small snack and R150 (~ USD 8) for their time. Interview recordings were transcribed, translated into English (from isiXhosa) where needed, and reviewed by a second team member for quality control.

### Data analysis

A subset of transcripts was read and open-coded by the study team using an inductive approach [69, 70]. Codes relating to TB illness experiences and mental and tangible resources, as defined by NIRM, were identified and consolidated into a codebook (Additional file 4). The codebook was then applied to all the transcripts by a qualitative research team using Dedoose (Version 9.0.17, Los Angeles, CA: SocioCultural Research Consultants, LLC), with coding iteratively assessed and discussed to ensure consensus and inter-coder reliability. Codes were subsequently organized into the following domains: (1) family and community environment, (2) TB symptom experiences, (3) clinic experiences, (4) mental and tangible resources that are either within one’s control (i.e., self-support) or sought/accessed/lost from others, (5) resiliency and vulnerability, (6) judgment (self/others), (7) discriminatory actions, and (8) the social impacts of TB and isolation.

Ongoing analysis was conducted throughout data collection and refined through a cyclic iterative process. Isolation constructs, gender, and the NIRM were used as a deductive interpretive lens to elucidate the relationships between and intersections of stigma, isolation, social networks, and the mental and tangible resources accessed, lost, or needed while participants were transversing the TB treatment journey [47–49]. Frequency analysis, memos, and pathway diagrams were leveraged to assess the relationships between mental and tangible resources during TB treatment. Familiarization and analytical memos focusing on TB isolation experiences, stratified by gender, were written including interpretive narratives and illustrative quotes. These memos were iteratively refined and discussed at weekly team meetings until data saturation was reached [71, 72]. Matrices to compare men’s and women’s TB illness experiences were developed through an inductive approach. Memos were also written to identify and refine findings as presented in matrices. Data analysis was conducted during data collection, identifying emerging codes and themes. To ensure a comprehensive representation of TB isolation and intersecting stigma experiences, all coded transcripts were systematically analyzed for TB-stigma experiences focusing on relevant domains and assessed for thematic consistency across genders. TB-stigma literature reviews and weekly study team discussions with study investigators further supported the confirmability and transferability of the findings and confirmed the interrelationships of TB stigma, social networks, and resource accessibility. The qualitative research team consisted of experienced study investigators specializing in public health and men’s health in South Africa, with existing relationships with Health District, none of whom were native South African or isiXhosa speakers. The qualitative research manager had expertise in HIV-related stigma research and health psychology, contributing to the study’s interpretive depth. This collective expertise informed the study design, data collection, and analysis while necessitating reflexivity in interpreting participants’ experiences. Various iterative memos and qualitative team discussions helped inform redundancy and guided against thematic bias. This was further supported by the representative sample of male and female participants based on sufficient individual experiences. The concepts of social isolation and social exclusion were then used to further help understand isolation experiences and pathways, and their dynamic relationship with stigma (i.e., anticipated, enacted and internalized). We further inductively examined changes in participants’ behaviors and social networks over time to determine how social networks were maintained, dissolved, and/or re-established as participants progressed along their illness-to-health journey.

### Participant representation

In-text quotes are attributed to participants by their study ID number, gender, and age. Quotes are presented verbatim, omitting non-essential sentences. Clarifications for the topic under discussion appear in [], and ellipses (…) indicate excerpts. Punctuation errors in the original transcription were reviewed and corrected to ensure accurate data integrity.

## Results

### Participant characteristics

We conducted IDIs with 142 men and women affected by TB with a median age of 37 (IQR 30–46) years (Table 1). Of those, 86 (60.6%) were men, 48 (33.8%) self-reported a previous history of TB, and 69 (48.6%) self-reported living with HIV. A larger proportion of men reported having a previous history of TB disease compared to women (37.2% vs. 28.6%). In comparison, a larger proportion of women reported living with HIV compared to men (67.9% vs. 36.1%), reflecting the gendered inequities and high HIV co-infection rates in South Africa. Compared to women, men were more likely to report being unemployed (72.1% vs. 58.9%) during TB treatment, living alone (29.0% vs. 16.1%) and being the primary household breadwinner (48.8% vs. 30.4%).

**Table 1:** Sociodemographic characteristics of interviewed participants (*N* = 142)

Characteristic (n/%)	Men (*n* = 86)	Women (*n* = 56)	Total (*N* = 142)
Age (median, IQR)	38 (31–48)	33 (27.5–44)	37 (30–46)
Relationship status			
Single	57 (66.3)	38 (67.9)	95 (66.9)
Married or living together	26 (30.2)	11 (19.6)	37 (26.1)
Divorced, separated, or widowed	3 (3.5)	7 (12.5)	10 (7.0)
Employment status			
Unemployed (actively or inactively looking)	62 (72.1)	33 (58.9)	95 (66.9)
Working for pay	7 (8.1)	6 (10.7)	13 (9.2)
Self-employed	7 (8.1)	4 (7.1)	11 (7.8)
Student	1 (1.2)	4 (7.1)	5 (3.5)
Other	9 (10.5)	9 (15.3)	18 (12.7)
Primary household breadwinner	42 (48.8)	17 (30.4)	59 (42.2)
Monthly household income < *R* 2 000 (~ $135)	58 (67.4)	34 (60.7)	92 (64.8)
Completed secondary education	20 (23.3)	18 (32.1)	38 (26.8)
Live alone	25 (29.0)	9 (16.1)	34 (23.8)
Had TB in the past			
Never	54 (62.8)	40 (71.4)	94 (66.2)
Yes, less than 2 years	11 (12.8)	3 (5.4)	14 (9.9)
Yes, more than 2 years	21 (24.4)	13 (23.2)	34 (23.9)
HIV status (self-reported)			
Positive	31 (36.1)	38 (67.9)	69 (48.6)
Negative	48 (55.8)	15 (26.8)	63 (44.4)
Not disclosed/Don’t know	7 (8.1)	3 (5.4)	10 (7.0)

### Symptom onset and judgment experiences

Both men and women described anticipating and experiencing stigma due to their TB status and clinic attendance. Visible weight loss was a particularly potent marker of TB illness that triggered negative comments and judgment.

Women expressed significant concern for being gossiped about. This often resulted from visible weight loss or behaviors perceived to be associated with TB disease:

I don’t like it when people talk about my weight, you know it makes me feel like there’s something I am not doing right, well in this case I knew that there’s something that I’m not doing right. [75, Female, 30 years]

I was thinking about what people will say [at the clinic]. People [would] be watching me when they pass by while I was sitting there [at the clinic]. I thought maybe they think I have TB because I drink a lot or smoke all those awful things like maybe they are judging me… [292, Female, 23 years]

These women reveal the connections between negative comments, causal attribution, and self-blame. Male patients also described their fear of negative comments, which they attribute to their weight loss and poor health:

P: I felt judged, and on my way to clinic people were looking at my weight. Some even asked why I have lost so much weight and that didn’t sit well with me.

I: Who did you feel was judging you? Other patients, clinic staff?

P: No, it was friends [87, Male, 37 years]

I get embarrassed. Maybe let’s say we are chilling at a certain place. Let me make an example. Let’s say I’m on my way to town. We’re sitting in a taxi with people, or any other place you can think of, or maybe I’m visiting a friend at his home, but any place I don’t normally go to, I do feel embarrassed like [in my mind, I think], ‘Ey. People will start asking questions here about my loss of weight’ and other things that I won’t be comfortable to talk about. So, I end up not going to these places because I’m ashamed and scared [41, Male, 35 years]

These quotes highlight men’s and women’s shared experiences of negative or unwanted comments about their weight loss and describe the psychological impact (e.g., shame, embarrassment, or self-blame) of having TB. Both men and women also anticipate being stigmatized as their TB-related symptoms become visible or as they start to attend clinic for their TB treatment. The fear of encountering questions or being forced into uncomfortable discussions about visible health changes led both men and women to avoid or not participate in certain social situations.

### Gendered pathways to isolation

Both women and men describe isolation as a manifest experience while ill with TB. However, women discuss enduring social isolation from social groups or community members, while men describe being excluded by others (i.e., social exclusion) such as other male peers.

#### Women: self-isolation and avoidance

Women often revealed how they refrained from venturing outside their homes due to the comments they received after exhibiting TB symptoms. These comments and unwarranted judgment predominantly emanated from their social networks and friends:

I: How were your symptoms affecting your social activities?

P: I used to be distant from people, even at school the way they treated me after [I was diagnosed]. I told them that this is not COVID, I have been diagnosed with TB. I was put in an isolated room to write my exams and during my lecture, the lecturer would sit outside when lecturing me…

I: Okay, I want to know during the time you were symptomatic and have not been diagnosed yet with TB.

P: Oh, everything was normal except with my friends, but I was able to talk with others who don’t know me. [248, Female, 39 years]

So, she [a friend] would say bad things about me. Then I would notice the mood, but some people would tell me straight up that “[friend’s name] is saying this and that about you”. I ended up avoiding them because they had bad attitude towards me. I isolated myself and lived in my shack with my boyfriend. [347, Female, 30 years]

A few men expressed concerns about anticipated negative comments from community members, particularly regarding the onset of symptoms such as weight loss and HIV associations that impacted their engagement with other men and women;

I: Was there a time when you wanted to keep your sickness a secret?

P: Yes brother, there was. I was someone who was always indoors people thought that I had left… Ey I was very slim… Yes, a lot brother, yoh a lot, so I thought [I should] hide myself… Because people would see that no A. [refers to self] was not like this, maybe he has AIDS or something, you see brother, I didn’t want to hear the bad things they would say.” [125, Male, 36 years]

P: As I discovered these symptoms, I became shy and distanced myself from friends and girlfriends. I focused on these symptoms.

I: How did these symptoms affect the way you interact with men in your life?

…

P: It affected me because I isolated myself from them [other men] because of my symptoms.

I: How did your symptoms affect your interactions with women in your life?

P: I did the same to women thinking they gossip or discuss about my symptoms when I am not around.” [99, Male, 39 years]

Most men were less explicit about deliberately concealing their condition in response to enacted stigma or changes in social networks but rather described being shy or ashamed of the onset of symptoms and thus not wanting to be seen by others whilst appearing ill of health. The anticipation of judgmental comments and actual negative remarks were more strongly emphasized as sources of discomfort and engendered self-consciousness among women. The latter participant [347, female, 30 years] closely observed shifts in the behavior and mood of others within their social networks, leading to avoidance of her friends. As a result of experienced mistreatment and altering social dynamics, several female patients conveyed feelings of shame and disappointment in friends that resulted in avoidant behaviors:

It made me very sad [when others noticed her weight loss]. I was ashamed I couldn’t even leave the house. People were talking. My neighbor (pointing to the right wall) was the first person to notice my weight loss. [116, Female, 31 years]

I even distance myself from people because they are no longer coming to visit me anymore and I told myself I will leave them.

I: So you told yourself to stay at home?

P: Yes.

I: How did you expect people to treat you after they noticed these symptoms?

P: At least friends were supposed to support each other, not judging before you know anything. Maybe they are judging because they have no knowledge, and they are scared.” [124, Female, 31 years]

This female patient [124, female, 31 years] described a desire for empathy and support from friends, instead of friends who are quick to judge or project fear due to their lack of knowledge. Overall, women described conscious decisions to distance themselves and/or self-isolate to shield themselves from adverse comments and community stigma.

#### Men: wanting to engage, but being excluded

Men often described how they were excluded from social interactions by friends once stigma markings became obvious or their TB status became known. Specifically, these men describe how friends would ‘distance themselves’ and exclude them from social events or community spaces due to their TB-related symptoms or illness. Several men use emotive language to describe how much these types of experiences impacted them:

…they were far from me. They didn’t even want to come and see me. Even when I take a walk during the day and go to them, they would not want me anywhere near them. Maybe if I had gone [there] with something to eat, they would not want to share with me. [125, Male, 36 years]

I had to stop a lot of things, and I was not able to go to places that I used to go [to]. Places I used to go and smoke at, and they [friends] used to distance themselves from me as if I was going to infect them. It was painful to me, but I saw where they were coming from [45, Male, 36 years]

Men who experienced social exclusion described continually trying to socially engage with friends but were constantly left out. They describe how their exclusion was driven by the fear of infection and how their exclusion exacerbated feelings of loneliness:

Like even today, some people treat me bad. Some people think the worst of me. That I can give them TB anytime because I cough and… I don’t know man. I just want to go and walk away from this place and not even worry about people anymore. Like living on my own strictly. I feel like that, I feel lonely. [15, Male, 38 years]

This man describes the emotional toll of being socially excluded and the pain and loneliness he experiences. They even expressed a desire to remove themselves from this situation due to this forced disconnection from others.

### The role of family in mitigating or exacerbating isolation

In general, several women and men described profoundly different experiences with their families when ill with TB. Specifically, women spoke of stable, reliable support from family members, while only men gave examples of friction with and hostility from their families and dissolution of romantic relationships.

#### Women: familial support and access to resources

Women seemed to often describe a high level of trust to disclose their TB illness to family members due to the perception that family does not gossip or divulge one’s illnesses in a negative manner. Familial acceptance was particularly important when women self-isolated to avoid community stigmatization:

My friends distanced themselves from me, so I was lonely. I was talking only with my family and since I was not the first person to get ill in the family, they were fine with it.

…

I: How did your symptoms affect your interactions with women in your life?

P: The women at my home they didn’t have a problem… outside I didn’t have anyone to talk to. [59, Female, 20 years]

I expected that they [household members] would just be upset and not talk about it, but they understood. I just trusted them, and I knew if I told a friend they would go around and divulge it in the streets. It is better to talk to the one at home [106, Female, 30 years]

These women describe the impacts of illness on social connections and the pivotal role their families played. Familial support was a crucial counterbalance to community stigmatization that could have otherwise discouraged or demotivated them during treatment:

She [my mother] said to me ‘my child, when you have TB people like to gossip. They like to say that 'oh [that] one has HIV.’ Then she said to me ‘don’t be scared, don’t be afraid. You will overcome this TB, you overcome it the first time you can overcome it the second time’… [12, Female, 30 years]

There are people who spread rumors. You have to tell yourself that you do not live for people. They cannot change who you are and there is nothing more important than your family. If you get support from your family, an outsider's support is not that important than your family’s support. The only thing that will make you to give up is if your family doesn't welcome you, but I think family is the most. [23, Female, 42 years]

In addition to family members providing mental and emotional support, women described how they continued to access tangible resources via familial networks:

I: Who supported you during this time?

P: My aunt

I: What type of support did she provide?

P: She gave me all the support I needed – money, advice, motivation, food, and shelter. She did everything for me.

…

I: Who else did you wish had given you support during this time?

P: No one except my friends. [59, Female, 20 years]

Okay when I found out that I had TB, I came home and told my family that I have TB. They didn’t judge me or distance themselves, but supported me and also changed the food we eat to healthy food for me. They did that so that I can quickly recover. [75, Female, 30 years]

These women highlighted the importance of their family networks in accessing tangible (e.g., money, food, and shelter) and mental (e.g., motivation, emotional support, companionship) resources crucial for combating loneliness and sustaining resiliency during their TB illness-to-health journey.

#### Men: friction, blame, and loss of familial support

In addition to being socially excluded by friends, a significant number of men gave examples of judgment and exclusion by their families described being judged and excluded by their families. There were however also several men who described support from partners or family members such as the following male participant, “My family was with me all the way [during the journey to complete treatment], they know my situation, they gave me the strength to handle the situation…” [185, Male, 28 years]. Similarly, the following male participant confirms social exclusion from peers and the necessary support during isolation,

My friends isolated themselves completely and people that motivated me were people here at home…. That is when I saw that when you have a problem there is no such thing as friends. When you have money it is all fun and games but when you start having a problem people isolate themselves [79, Male, 32 years]

Although women reported feeling increased irritability themselves and in the company of others due to ill-health, they did not report feeling excluded by family. Judgment towards men often arose from behaviors perceived to be related to contracting TB (e.g., drinking alcohol, smoking, partying). Furthermore, several men described strained or dissolution of romantic relationships while ill with TB. These strained familial and romantic relationships revealed significant implications for men when there was limited support:

After those [TB] symptoms, it was hectic at home, especially [with] my mom and uncles. My mom is short-tempered and when she would scold me, she would use that I have TB against me and say that I bring over friends that have TB

[326, Male, 46 years]

It is painful on my family’s side only because my mother was someone who loved [me]. But when I had TB, I have never seen her, she doesn’t even call. And my sister is the one who changed when she found out that I have TB. She [sister] didn’t give any [support] except the ones I told you, and the cereals she bought me once. She never bought them, and she never came again to check up on me.

Eh! I wish my sister, and my mother would have supported me, because when I was working, I was doing everything for her, my mother. Now she is living well in the villages, and she has money, but I am not looking for her money, but I wanted her to support me especially with food because I was really struggling with getting food, because I was not working, and my girlfriend was not working as well. [138, Male, 38 years]

These men described how their families accused them of putting them at risk for TB, distanced themselves, and stopped providing them with any type of support. Men further explained how retention of familial support would have helped them overcome existing challenges, including food and economic insecurity.

Familial judgment and exclusion also engendered self-blame for having TB. In extreme cases, though not unique, several men described being physically locked in rooms by family members due to perceived safety concerns. This deprived these men of basic needs such as access to a bathroom:

…I decided to move back to my shack and my elder brother he was just giving me the bad look when I [would] walk with friends. But [he] asked me ‘why [do] you have this [TB]?’ and I said it[s] because of alcohol, walking at night, cigarette and weed…

My sister chased me to stay in my room and close[d] the door because of [my] coughing sometimes. I wanted to go to the toilet, but I [couldn’t] because my door was locked. So, I [would] just open [a] window and take out my penis or I take [a] bucket, so I didn’t like [these] things [199, Male, 31 years]

Interestingly, only men spoke about the dissolution of their romantic relationships because of their illness. Although women had relationships, no relationship dissolution was reported because of their TB during the interviews. Only one female participant feared that their partner would leave them, more likely exhibiting anticipated isolation fears for having TB rather than actual enacted discrimination or relationship dissolution:

I thought he would leave me, I was hopeless, but I remained strong, I thought that since I am sick he would leave me and I will be alone, but he didn’t [leave me]. [238, Female, 39 years]

This participant goes on to describe tangible and mental support from their boyfriend, mentioning that “… he provides me with everything I need, food and money. Even when it is my appointment date he would remind me to wake up and go to the clinic, he always encourages me.” [238, Female, 39 years].

The following men describe the end of their marriage or relationship with a girlfriend:

…at the clinic, my wife’s sister was a nurse and heard I was taking my [TB] treatment at [another clinic]. She said she knows I am taking treatment, and I can no longer be with her sister. And I ended up losing her [wife], and she said she no longer wants marriage. It really hurt me. [237, Male, 40 years]

…Ey brother, I lost most of them [women] because of my health… she [girlfriend] did not notice that I am not okay, but she heard rumors, she distanced herself. I was not okay, I tried to explain but she already heard a lot, it was [too] late for me

[7, Male, 26 years]

Social losses and ill-health also translated into internalized negative feelings and emasculation as described by this patient:

…[interacting] with men I don't know what to say because I don't even feel like a man anymore. I feel too weak to be a man because I can't even provide for myself. How would a man like me provide for a woman? That's why I don't even have a girlfriend anymore because I know that I can't even provide for myself.

[15, Male, 38 years]

Some men described how their TB illness affected their physical strength and sexual performance and attributed these factors to the dissolution of their romantic relationships. Overall, strained familial relationships or loss of romantic partners exacerbated these men’s isolation experiences and drastically reduced their ability to access the necessary resources and support they needed when ill with TB.

### Regaining health and re-establishing (altered) social networks

Most patients described feeling better within three months after initiating TB treatment. This was often associated with increased appetite and an improved sense of being: “I felt better after two months, and on the third month I could notice I was better now. I was eating too much day and night”. [83, Male, 47 years] Improved health and strength were associated with both men and women re-establishing connections with those who had socially excluded them. However, for some, these connections were irreparably altered.

Both men and women described social re-engagement once others perceived or observed them to have regained health. However, concerns lingered about the stability and nature of these re-established connections:

…they [friends at college] were still acting funny, but it took them a long time to accept me, until they saw that I was gaining back my weight. Even though I showed them my clinic book, they still had doubts at first [248, Female, 25 years].

I lost a lot of people; they thought I had COVID so they were protecting themselves.

I: How did all this make you feel?

P: It made me feel sad. It’s not like I’m the only person with TB around here. So, I didn’t understand why they were treating me this way.

I: And when you got better, how did people treat you?

P: I went back to work right after I felt stronger. And after that, everything went back to normal. You know how people are when they see that you can work again and make money [87, Male, 37 years]

These individuals experienced initial hesitation from others, which persisted despite presenting clinical information to friends. Re-establishment of social networks took time and only happened once they regained their strength and resumed their roles in society. Unfortunately, other men and women described either a lack of agency to reconnect with lost relationships, or an active decision to reject those that were not there when they needed them:

P: …no I cut it off with all my friends. I cut them off…

I: What stopped you from discussing that you got TB with your friends?

P: Because they couldn’t help and [weren’t] motivating me [14, Male, 32 years]

The ones that isolated me are still isolating me. In the community I no longer have friends. People I was friends with disappeared, also at church and the ones I see are the ones I did not go to church with. I was visited by the ones from Thursday service instead of the Saturday service. [207, Female, 56 years]

P: They see you as a weak person

I: You also mentioned that you do not have friends anymore

P: Yeah, nobody comes to me anymore –none of my friends. [15, Male, 38 years]

Lack of support or motivation during illness led some individuals to sever ties with absent friends. Others described feeling abandoned even when they regained their health. Regardless of whether original connections were permanently altered, re-established, or lost, both men and women had to actively navigate or redefine their social connections. These experiences highlight the long-term social sequelae of TB, even when someone has successfully completed their treatment or regained their health.

## Discussion

**Fig. 1:**
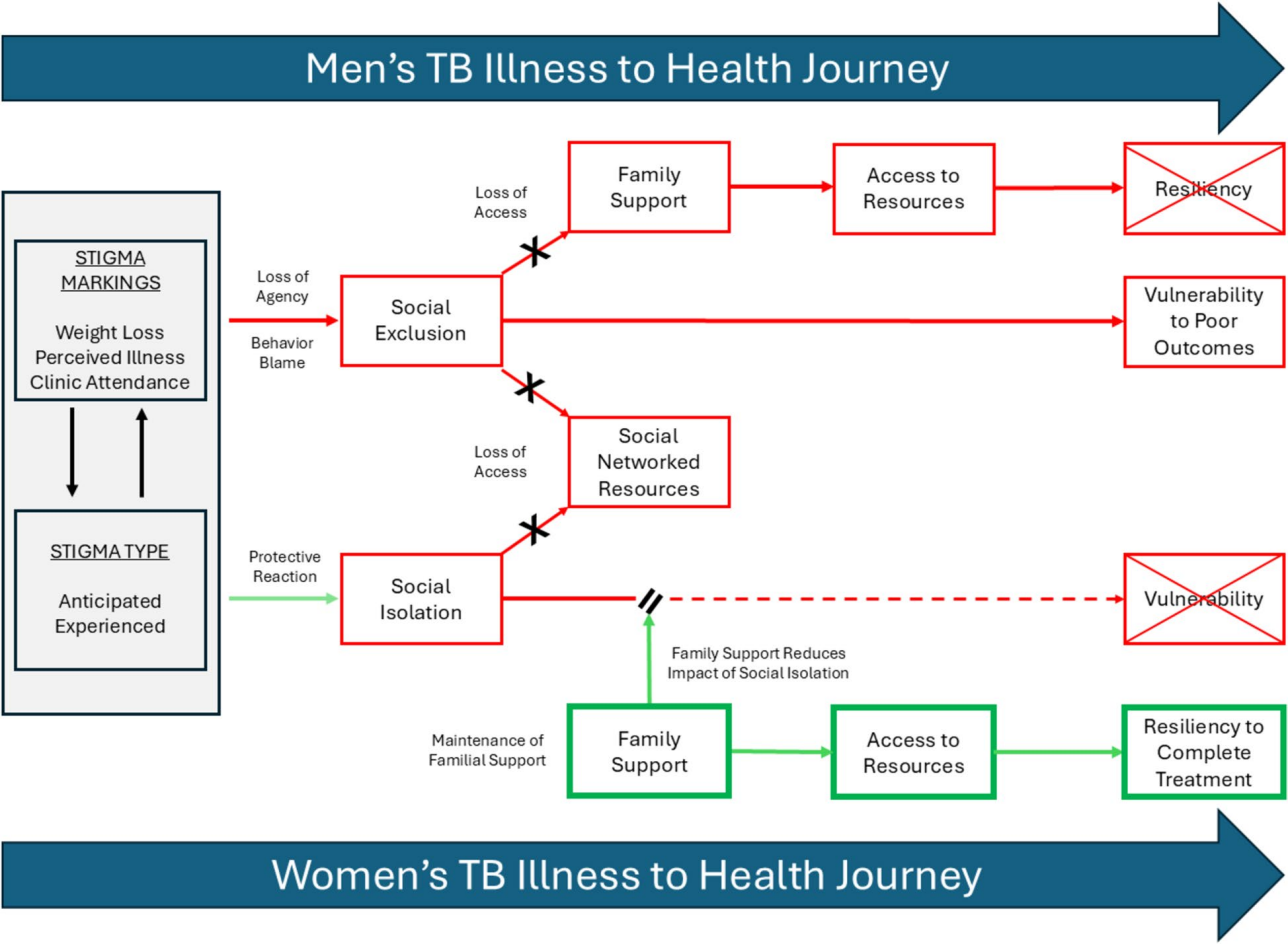
Gendered pathways and impact of stigma and isolation men and women’s health journeys when ill with TB in the BCM Health District, Eastern Cape, South Africa

Our findings substantiate a broader body of literature describing the ubiquity of TB-related stigma, with isolation being a manifest experience of stigma [4, 10, 14, 15, 73]. TB-related stigma can include shame, judgment, fears, financial limitations, and marital constraints. Men can experience stigma due to weakened strength and loss of status due to financial constraints, linked to unhealthy masculine norms, while women are reported to face more prejudice and familial stigma, with greater psychosocial impacts [10, 14, 15]. HIV and TB stigma share similar drivers, including fear of infection, economic impact, and social constructs that lead to rejection or isolation [16, 74, 75]. In South Africa, high co-infection rates have been well-documented and highlight the intersectionality of HIV and TB stigma [43, 45, 75]. However, there has generally been limited exploration or mapping of the pathways through which isolation occurs or functions during TB illness and treatment as a result of TB-related stigma. Experiences of social isolation and social exclusion are reported in the TB literature [7, 17, 18, 21, 56, 76], such as self-isolation for judgment fears or concealing household TB status to avoid criticism, and social exclusion, which can limit economic opportunities [10, 15]. Our study conducted amongst men and women with TB treatment experience within the BCM health district is the first to describe and delineate distinct, gendered pathways among a South African population by which TB stigma facilitates social isolation or social exclusion. Figure 1 delineates the main findings from our study mapping these isolation experiences by gender. Further, these findings may be similarly found in high TB-burdened and under-resourced settings. By applying the NIRM in conjunction with isolation concepts and stigma types, we further describe how familial networks mediated access to resources for these men and women, and in turn, mitigated or exacerbated their isolation [77, 78]. In this context, we show how families are able to promote women’s resiliency while magnifying men’s vulnerability when ill with TB.

In our study, men and women experienced distinct pathways towards isolation in response to TB stigma (Fig. 1). Both men and women reported that observable weight loss or declining health was the main trigger for their anticipated and experienced stigma, which has often been associated with HIV [75, 79]. However, women described withdrawing more quickly from their social networks, while men continued seeking social interactions until they were forcibly excluded. Specifically, men reported being socially excluded by their peers due to fear of infection and by family members due to judgment and behavior blame. In contrast, women mentioned engaging in social isolation as a protective measure against negative comments and judgments from others. Previous research in South Africa found that women fear anticipated stigma, feel the need to self-isolate due to TB illness, and experience community discrimination. At the same time, men exhibited more internalized and enacted stigma linked to associated TB-related risk behaviors such as socializing and substance use, and reported reduced potential for financial contribution [15, 19]. In our study, men were more likely to lose their social network status. These findings highlight gendered differences in how men and women are impacted by and respond to stigma, with women exhibiting a protective response and men losing agency.

Isolation can hamper engagement and status from social networks including financial opportunities [4, 15]. Familial networks and support have been well described as crucial to promoting resiliency during one’s illness-to-health journey including adherence and quality of life [37, 39, 62, 77, 78, 80, 81]. Networks can for instance provide resources that prevent or overcome stigmatization (e.g., healthy foods for increased treatment intake and regained strength or support for internalized stigma reduction) and ensure resiliency to cope with illness while social programs are limited [47, 62]. In our study, based on women’s narratives, their familial networks emerged as a crucial buffer against social isolation by other social network members, by providing emotional support and material resources, thus helping them cope with and navigate their TB illness and treatment, and facilitating resiliency (Fig. 1) [39, 62, 78]. This may flow from the fact that in high HIV burden communities, women act as caregivers, head households, and economic contributors to their families, and to further avoid increased vulnerability among female caregivers, families may reinforce their importance within the family structure and encourage support when ill [40, 78, 82–84]. Furthermore, South African women often reported relying on strong female networks, particularly in households where men migrate for work, offering them a buffer during illness [39, 82–84]. This contrasts with studies from Southeast Asia, where women experience significant social exclusion, neglect from family members, and rejection by husbands, reflecting a combination of cultural, social, and economic factors [7, 14, 18, 85]. In India and Bangladesh, traditional gender norms often render women more dependent on male family members, leading to neglect or exclusion when they cannot fulfil domestic roles [86–88]. In these countries, TB-related stigma often intersects with marital and reproductive expectations, causing women to be rejected by their husbands or in-laws [18, 76, 88, 89]. Furthermore, women in Southeast Asia, who are often part of joint family systems and experience existing gender inequalities, may be more vulnerable to rejection when TB is seen as a financial or social burden [37, 89, 90]. Overall, these contextual factors highlight why familial support for women with TB may differ significantly between South Africa and Southeast Asian countries and gender norms and disparities intersect with TB experiences differently.

Men in our study describe experiencing overt rejection and social exclusion by both peers and family, resulting in distinct challenges and resource insecurity while ill with TB (Fig. 1). Their social exclusion experiences are likely a result of gender norms around masculinity and health, where illness is equated to weakness and seeking help is perceived as a threat to their social status [13, 43, 91]. For instance, as a result, men may continue to “endure” their symptoms and delay seeking care due to social norms, worsening their health, and risking social exclusion due to reduced network contributions and infection fears. This may also explain their heightened expressions of shame or loneliness [13, 47]. Similarly, social exclusion compels individuals to seek avenues for reintegration or social inclusion, which may explain why men persist in pursuing peer interactions despite experiencing illness and exclusion [52]. This exclusion extended to romantic relationships, where men reported experiencing estrangement or dissolution [14, 24], a dynamic not reported by any women in our study. Men in sub-Saharan Africa previously spoke more of relationship dissolution due to TB illness and the impact on socializing and sexual relations [13, 43]. The lack of cited trust and support from female caregivers by several male participants, a crucial element for treatment adherence, further exacerbated men’s exclusion experiences, leaving them with diminished access to essential mental and tangible resources [13]. Consequently, men exhibiting prioritization of work over health, often framed as a negative aspect of masculinity [11, 13, 43, 92], may instead represent a survival strategy to retain social and economic resources that they know could be lost or difficult to re-establish, thus compromising their survival during and after illness. This may also explain why men, unlike women, resisted self-isolation in response to community stigmatization, as maintaining their social status within existing social and family networks preserves access to necessary resources [10, 15, 24]. The differences in how men and women navigate stigma in this context may also reflect their respective gendered roles in accessing and contributing to social networks. Further, a reported loss of support from friends for individuals living with TB can exacerbate stigma experiences and reduce resiliency during care [93]. Specifically, while women are shown to benefit from relatively stable and enduring familial networks, men seem to rely more on broader, yet often more fragile, peer networks that are more easily disrupted by illness [47, 82, 94, 95]. As a result, when illness undermines men’s ability to meet social expectations or contribute to these networks, they are more likely to experience social exclusion, social status losses, and a resultant loss of resources [47, 96]. Peer support or coaching programs for men could help bridge this gap by providing alternative resource networks and challenging masculinity norms and internalized stigma that impact health, ultimately reducing the risk of loneliness [4, 23, 81, 97, 98].

Both men and women in our study described a gradual reintegration into social networks as their health improved, but this reintegration was neither immediate nor universally successful. The findings suggest that the impacts of social exclusion and isolation extend beyond the symptomatic phase of TB, with some patients facing sustained social challenges even during and after treatment. This persistent exclusion has been previously reported in TB-related stigma literature [7] and highlights the need for ongoing support during the recovery phase to facilitate reintegration into social networks and mitigate long-term stigma effects.

Considering both the strengths and limitations of this study, our use of an extensive qualitative dataset (142 in-depth interviews), representing a diverse range of experiences along the entire TB care cascade within the South African context, allowed for a more comprehensive exploration of the nuanced experiences related to TB stigma, and the gendered pathways to isolation and dynamics of accessing resources that have been previously under-researched. The NIRM framework’s ecological approach highlights how networks influence health behaviors and coping by exchanging mental and tangible resources according to need on varying levels. NIRM however does not unpack the psychological processes of stigma and which resources would make the most impact on TB-related health behaviors. Instead, the framework incorporates stigma as a disruptor of networks, offering a valuable lens to explore how social isolation and exclusion impact resource access and vulnerability in men and women on TB treatment [47]. Further, the findings underscore socio-cultural factors such as social and gender roles, family structures, and socioeconomic status, highlighting the complex interplay of gender norms, stigma, and social determinants that differ between countries and global regions. During analysis, participants’ HIV status and prior TB experiences were considered in relation to TB due to high co-infection rates [45, 75]. However, we found no distinct differences in reported TB-related stigma based on HIV, and a limitation of the study was that this variable was self-reported. To explore gendered experiences of TB-related stigma, we did not stratify findings by age, which could have further enhanced our understanding of intersectionality. Although relationship dissolution and familial tension have been previously reported by women in the TB literature, our sample of female participants did not report these experiences related to TB illness during the IDIs. While the cross-sectional design offers a snapshot of the emergent findings related to the impact and lingering effect of stigma, a deeper exploration is needed to understand these psychological effects and how stigma is experienced across the TB trajectory. Future research using longitudinal or serial interviews could further illuminate how relationships with family and social members and the interchange of mental and tangible resources evolve over time.

## Conclusions

Findings from this study reveal women’s and men’s distinct and gendered experiences of social isolation and exclusion, respectively, during TB illness that impact access to key resources for treatment support [4, 30, 32, 73, 96]. Furthermore, it highlights the social and economic ramifications of TB illness for both men and women, as well as the context-specific experiences of TB-related stigma in South Africa as compared to other global regions. Men’s weaker peer networks and focus on survival due to socio-economic pressures, along with women’s felt need to self-isolate due to illness and fear of judgment, jeopardize access to essential resources during treatment. This work underscores the critical role of familial networks in buffering or exacerbating the impact of stigma on individuals’ TB journeys, well-being, and health outcomes. These findings can inform responsive or differentiated interventions to mitigate the health impact of TB stigma, recognizing gender-specific needs [10]. For instance, peer support programs for men [23, 87] and family-centered support models for women [47] can provide tailored resources, foster social connections, and reduce stigma-related barriers to care.

## Supplementary Information

Additional file 1. Native Language Abstract.

Additional file 2. Consolidated criteria for reporting qualitative studies (COREQ): 32-item checklist for interviews and focus groups.

Additional file 3. Interview Protocol.

Additional file 4. Final Codebook.

## Data Availability

The datasets produced or analyzed in this study are not publicly accessible due to the confidential nature of the interview responses and transcripts, which may contain identifying information based on participant responses. In accordance with South Africa's Protection of Personal Information Act (POPI Act), there are restrictions on publicly sharing this data. Data can be shared upon reasonable request from the corresponding author.

## Abbreviations

BCM: Buffalo City Metropolitan Health District
DS-TB: Drug-susceptible tuberculosis
IDI: In-depth interview
LTC: Loss to care
MTRs: Mental and tangible resources
NIRM: Network-Individual-Resource Model
PLWTB: People living with tuberculosis
