# Supplementary material for: Social isolation, social exclusion, and access to mental and tangible resources: mapping the gendered impact of tuberculosis-related stigma among men and women living with tuberculosis in Eastern Cape Province, South Africa
Source: BMC Glob Public Health. 2025 Jun 5;3:50. doi: 10.1186/s44263-025-00166-6 (PMC12142910; doi:10.1186/s44263-025-00166-6)
Supplement: Supplementary file 1 — Additional file 1. Native Language Abstract. [file 44263_2025_166_MOESM1_ESM.docx]

This translation in isiXhosa was submitted by the authors and we reproduce it as supplied. It has not been peer reviewed. Our editorial processes have only been applied to the original abstract in English, which should serve as reference for this article. This translated abstract is published under the same license as the article.

**ISIXHOSA ABSTRACT (UMXHOLO)**

**Imvelaphi**: Ngo-2022, baqikelelwa kwi-10.6 yezigidi zabantu abanesifo sephepha kwihlabathi jikelele, amadoda athwele umthwalo omkhulu wesifo xa kuthelekiswa nabasetyhini. EMzantsi Afrika ngokukodwa, amadoda afumana ingozi ephezulu yeziphumo ezibi kunye nokufa okunxulumene ne-TB kunabasetyhini. Ukubekwa ibala nokubekelwa bucala kwabantu abaphila nesifo sephepha kubhaliwe kakukhulu ngayo. Iindlela zesini apho ibala elinxulumene ne-TB likhokelela ekubeni umntu abekelwe bucala okanye libe neempembelelo zokufikelela kwimithombo yoncedo ngexesha lokugula komntu ukuya empilweni aziqondwa kakuhle.

**Iindlela:** Sibe nodliwano-ndlebe ne-PLWTB efumana unyango kwiiklinikhi zikarhulumente eBuffalo City Metro District Health, kwiPhondo leMpuma Koloni, eMzantsi Afrika. Izikhokhelo ezinesiqingatha zakhiwe zijonge: Amava eempawu ze-TB; ukufikelela-kukhathalelo; inkuthazo yonyango; abaxhasi abaphambili; kunye nokufikelela kwimithombo yengqondo, kunye nezinto ezibonakalayo (MTRs) ngexesha lokugula. Ukhowudo oluvulekileyo lwenziwe ngendlela esebenzisa imigqaliselo ethile ukuze kufikelelwe kwisigqibo ngokubanzi, kunye nemimandla ye-MTR eyaziswa yi-Network-Individual-Resource Model. Iziphumo zahlalutywa ngenkqubo yomjikelo, ephinda-phindwayo kunye neyokuxhuzula kusetyenziswa ukubekwa bucala kwentlalo kunye nokungabandakanywa njengeelensi zokutolika. Iimemo kunye nemephu yendlela ivavanye umahluko ngokwesini kwibala, ukwahlukaniswa, kunye nokufikelela kwii-MTRs zothungelwano.

**iziphumo:** Ikhulu elinamashumi amane anesibini e-PLWTB (Amadoda=86; Abasetyhini=56) abathathe inxaxheba kudliwano-ndlebe. I-PLWTB ichaze ibala le-TB elixhaphakileyo kunye nokubekelwa bucala. Abasetyhini bachaze ukuzibekela bucala eluntwini ukuphendula kwibala elimiselweyo nelilindelweyo. Amadoda achaze ukukhutshelwa ngaphandle okuseqhubekayo ngabahlobo kunye nosapho. Ukugcina kwabasetyhini ubudlelwane bosapho kuququzelele ukufikelela kwii-MTRs ngelixa egula. Ukukhutshwa okucwangcisiweyo kwamadoda (umzekelo, ngabom okanye ukunyanzelwa ngoontanga okanye usapho) kunciphise i-arhente yabo ukufikelela kwizibonelelo. Amadoda nabasetyhini bachaze ukuphinda bafumane amandla omzimba kunye nokuchacha kwinethiwekhi yoluntu ngonyango, kodwa kunye neempembelelo ezizinzileyo zokubekwa ibala emva konyango.

**Isiphelo:** Sifumanise iindlela zesini apho ukubekwa ibala kwe-TB kunye nokubekelwa bucala kuchaphazela ukufikelela kwii-MTRs. Kwabasetyhini, ukubekwa ibala kukhokelela ekubeni babekelwe bucale ekuhlaleni, kodwa uthungelwano lwentsapho lwanceda ukugcina ukufikelela kwi-MTRs, ukukhuthaza ukomelela. Amadoda afumene ukukhutshelwa ngaphandle kwentlalontle, ukunciphisa amandla ukufikelela kwi-MTRs, kunye nokwanda komngcipheko ngexesha lokugula. Iziphumo zingakhokela ungenelelo oluphendula ngokwesini ukunciphisa impembelelo yebala le-TB kwiziphumo zempilo.
