# Supplementary material for: Social isolation, social exclusion, and access to mental and tangible resources: mapping the gendered impact of tuberculosis-related stigma among men and women living with tuberculosis in Eastern Cape Province, South Africa
Source: BMC Glob Public Health. 2025 Jun 5;3:50. doi: 10.1186/s44263-025-00166-6 (PMC12142910; doi:10.1186/s44263-025-00166-6)
Supplement: Supplementary file 2 — Additional file 2. Consolidated criteria for reporting qualitative studies (COREQ): 32-item checklist for interviews and focus groups. [file 44263_2025_166_MOESM2_ESM.docx]

**Consolidated criteria for reporting qualitative studies (COREQ): 32-item checklist for interviews and focus groups**

Developed from: Tong A, Sainsbury P, Craig J. Consolidated criteria for reporting qualitative research (COREQ): a 32-item checklist for interviews and focus groups. International Journal for Quality in Health Care. 2007. Volume 19, Number 6: pp. 349 – 357

| Item No. | Topic | Guide Questions/Description | Reported on  Page no. |
| --- | --- | --- | --- |
| Domain 1: Research team and reflexivity | | | |
| *Personal Characteristics* | | | |
|  | Interviewer/  facilitator | Which author/s conducted the interview or focus group? | **Methods, pg. 8**  “Interviewers were South African native isiXhosa speakers with public health or social work experience, and knowledge of the health district that enabled them to engage participants in a culturally sensitive manner.” Interviewers were supervised by authors LDV and JD. |
|  | Credentials | What were the researcher’s credentials? E.g., PhD, MD | AMM: PhD, MPH  LDV: MSc  JD: PhD |
|  | Occupation | What was their occupation at the time of the study? | AMM: Co-Principal Investigator and Site Lead  LDV: Qualitative Research Manager  JD: Co-Principal Investigator |
|  | Gender | Was the researcher male or female? | AMM: Male LDV: Female JD: Male |
|  | Experience and training | What experience or training did the researcher have? | The research team has extensive expertise in public health research in South Africa over the past decade, including tuberculosis and men’s health. |
| *Relationship with participants* | | | |
|  | Relationship established | Was a relationship established prior to study commencement? | Researchers had no relationships with participants. Interviewers were not facility-based during recruitment. |
|  | Participant knowledge of the interviewer | What did the participants know about the researcher? E.g., Personal goals, reasons for doing the research | **Methods, pg. 6**  The reason for the research was introduced by research assistants and explained using an informed consent form. Similarly, each interview began with an introductory script. |
|  | Interviewer characteristics | What characteristics were reported about the interviewer/ facilitator? E.g., Bias, assumptions, reasons, and interests in the research topic | **Methods, pg. 8**  “Interviewers were South African native isiXhosa speakers with public health or social work experience, and knowledge of the health district that enabled them to engage participants in a culturally sensitive manner. Interviewers received Good Clinical Practice training from management staff before study implementation, and Interviewing Skills training from qualitative research experts before conducting the in-depth interviews.” |
| Domain 2: Study Design | | | |
| Theoretical framework | | | |
|  | Methodological orientation and theory | What methodological orientation was stated to underpin the study? e.g., grounded theory, discourse analysis, ethnography, phenomenology, content analysis | **Methods: Study framework, pg. 5**  **“…**This study was guided by the Network-Individual-Resource Model (NIRM), and concepts of isolation and stigma.[46–49]” |
| Participant selection | | | |
|  | Sampling | How were participants selected? e.g., purposive, convenience, consecutive, snowball | **Methods, pg. 7**  “Participants were prospectively followed and purposively invited for an in-depth interview (IDI) based on their progression along the TB treatment cascade (i.e., currently on treatment; missed ≥15 days of a treatment refill visit; recently completed treatment).” |
|  | Method of approach | How were participants approached? e.g., face-to-face, telephone, mail, email | **Methods, pg. 7**  “All eligible individuals (adult men and women) were invited to learn about the study, consecutively recruited, consented and administered a study questionnaire by study staff at time-of-enrollment.” |
|  | Sample size | How many participants were in the study? | **Methods, pg. 7**  “…Given South Africa's high TB burden, the study aimed to recruit up to 30 participants of each gender at each stage of the TB care cascade to ensure a sufficiently representative sample stratified by gender and treatment experience. This approach also sought to better understand gender-centered TB care preferences. Participants were those successfully contacted by interviewers and willing to participate in an IDI.” |
|  | Non-participation | How many people refused to participate or dropped out? Reasons? | Not applicable. |
| Setting | | | |
|  | Setting of data collection | Where was the data collected? E.g., home, clinic, workplace | **Methods, pg. 8**  “Interviews were scheduled with participants either telephonically or via home visits. Interviews were conducted in a participant’s preferred language (English or IsiXhosa), and in a private location agreeable to the study participant (i.e., within the comfort of their own home).” |
|  | Presence of nonparticipants | Was anyone else present besides the participants and researchers? | Field notes indicate that, on occasion, other household members were present during the interview. However, their presence was contingent upon the participant's approval and comfort. In-depth interviews were generally encouraged to be conducted one-on-one with the interviewer in a private and quiet setting. This was also discussed and confirmed with the interviewer during the scheduling process. |
|  | Description of sample | What are the important characteristics of the sample? e.g., demographic data, date | **Methods, pg. 7**  “…“…Given South Africa's high TB burden, the study aimed to recruit up to 30 participants of each gender at each stage of the TB care cascade to ensure a sufficiently representative sample stratified by gender and treatment experience. This approach also sought to better understand gender-centered TB care preferences. Participants were those successfully contacted by interviewers and willing to participate in an IDI.” |
| Data collection | | | |
|  | Interview guide | Were questions, prompts, guides provided by the authors? Was it pilot tested? | **Methods, pg. 7-8**  “Semi-structured interview protocols were developed to examine the following domains: experiences and perspectives of TB symptoms; access to clinical care and services; individual motivations for treatment; disclosure decision-making; social and familial network support during TB illness; perspectives of other men and women’s TB experiences; and probing for tangible and mental resources accessed during their TB illness and treatment journey. Open-ended questions in line with these domains further explored changes in the behaviors and dynamics of their social and familial networks, experiences with anticipated, enacted and internalized stigma, judgment, the ability to access support, perceived impacts of their illness on their lifestyle and relationships, and experiences of isolation and network membership exclusion (Additional File 2).” |
|  | Repeat interviews | Were repeat interviews carried out? If yes, how many? | Not applicable |
|  | Audio/visual recording | Did the research use audio or visual recording to collect the data? | **Methods, pg. 8**  “Interview recordings were transcribed, translated into English (from isiXhosa) where needed, and reviewed by a second team member for quality control.” |
|  | Field notes | Were field notes made during and/or after the interview or focus group? | Field notes were collected shortly after the interview. |
|  | Duration | What was the duration of the interviews or focus group? | Interviews were audio-recorded and lasted approximately 60-90 minutes. |
|  | Data saturation | Was data saturation discussed? | **Methods, pg. 9**  “Familiarization and analytical memos focusing on TB isolation experiences, stratified by gender, were written including interpretive narratives and illustrative quotes. These memos were iteratively refined and discussed at weekly team meetings until data saturation was reached [71,72].” |
|  | Transcripts returned | Were transcripts returned to participants for comment and/or correction? | Transcripts were not returned to participants but underwent a thorough review process, including a second reviewer and additional check as conducted by the project coordinator. |
| Domain 3: analysis and findings | | | |
| Data analysis | | | |
|  | Number of data coders | How many data coders coded the data? | **Methods: Data analysis, pg. 8**  “The codebook was then applied to all the transcripts by a qualitative research team using Dedoose (Version 9.0.17, Los Angeles, CA: SocioCultural Research Consultants, LLC), with coding iteratively assessed and discussed to ensure consensus and inter-coder reliability.” |
|  | Description of the coding tree | Did authors provide a description of the coding tree? | A subset of transcripts was read and open-coded by the study team using an inductive approach [69,70]. Codes relating to TB illness experiences and mental and tangible resources, as defined by NIRM, were identified and consolidated into a codebook (Additional File 3). The codebook was then applied to all the transcripts by a qualitative research team using Dedoose (Version 9.0.17, Los Angeles, CA: SocioCultural Research Consultants, LLC), with coding iteratively assessed and discussed to ensure consensus and inter-coder reliability. Codes were subsequently organized into the following domains: 1) family and community environment; 2) TB symptom experiences; 3) clinic experiences; 4) mental and tangible resources that are either within one’s control (i.e. self-support) or sought/accessed/lost from others; 5) resiliency and vulnerability; 6) judgment (self/others); 7) discriminatory actions and 8) the social impacts of TB and isolation.” |
|  | Derivation of themes | Were themes identified in advance or derived from the data? | **Methods: Data analysis pg. 9**  “Ongoing analysis was conducted throughout data collection and refined through a cyclic iterative process. Isolation constructs, gender and the NIRM were used as a deductive interpretive lens to elucidate the relationships between and intersections of stigma, isolation, social networks, and the mental and tangible resources accessed, lost or needed while participants were transversing the TB treatment journey.” |
|  | Software | What software, if applicable, was used to manage the data? | Dedoose (Version 9.0.17, Los Angeles, CA: SocioCultural Research Consultants, LLC) |
|  | Participant checking | Did participants provide feedback on the findings? | Not applicable |
| Reporting | | | |
|  | Quotations presented | Were participant quotations presented to illustrate the themes / findings? Was each quotation identified? E.g. Participant number | **Methods: Participant representation pg. 10**  “In-text quotes are attributed to participants by their study ID number, gender, and age. Quotes are presented verbatim, omitting non-essential sentences. Clarifications for the topic under discussion appear in [ ], and ellipses (…) indicate excerpts. Punctuation errors in the original transcription were reviewed and corrected to ensure accurate data integrity.” |
|  | Data and findings consistent | Was there consistency between the data presented and the findings? | **Methods pg. 9-10**  “The concepts of social isolation and social exclusion were then used to further help understand isolation experiences and pathways, and their dynamic relationship with stigma (i.e., anticipated, enacted and internalized). We further inductively examined changes in participants’ behaviors and social networks over time to determine how social networks were maintained, dissolved, and/or re-established as participants progressed along their illness-to-health journey. |
|  | Clarity of major themes | Were major themes clearly presented in the findings? | Findings and participant quotes have been presented under major themes/headings in the results |
|  | Clarity of minor themes | Is there a description of diverse cases or discussion of minor themes? | Not applicable |
