# Supplementary material for: Social isolation, social exclusion, and access to mental and tangible resources: mapping the gendered impact of tuberculosis-related stigma among men and women living with tuberculosis in Eastern Cape Province, South Africa
Source: BMC Glob Public Health. 2025 Jun 5;3:50. doi: 10.1186/s44263-025-00166-6 (PMC12142910; doi:10.1186/s44263-025-00166-6)
Supplement: Supplementary file 3 — Additional file 3. Interview Protocol. [file 44263_2025_166_MOESM3_ESM.pdf]

INTERVIEW PROTOCOL

Introduction Script

Good morning/afternoon. My name is ____________________________ and I work for the Foundation for Professional Development (FPD). We would like to speak and interview you as part of a study we are doing with the Buffalo City Metropolitan (BCM) Department of Health. We are doing research on tuberculosis (TB), which is very common throughout South Africa, and especially in Eastern Cape. Our study is being done with men and women, such as yourself, who have been ill with TB. Specifically, we are trying to understand people's experiences while ill with TB. When answering, it is important that you respond as clearly and with as much detail as possible. For example, [interviewers will share a short anecdote demonstrating the level of detail desired]. Your answers will be kept anonymous to protect your privacy. We are grateful for your time and openness in answering our questions. Your knowledge, experiences and insights will be extremely helpful as we try and develop better ways of supporting people with TB.

In some of the following question, it may seem that I am asking you something that you may have already told me about. We do this because we are trying to deeply understand your experiences with TB. What may seem to you as a repeated story or answer is to us a treasure that can really help us learn about and hear your story.

Social & Familial Network, Support and Resources *[First, I'm curious to learn a bit about you and the people in your life, such as your family, those who you live with, and your friends. Is that ok?]*

1. What makes your community health?
2. What makes your community unhealthy?
3. Can you tell me an important or memorable story, either positive or negative, about an experience you had with a family member or friend that influenced your health (physical, mental)?
    a. Tell me why this story is important to you?
    b. How does this story affect how you think about your health?
4. Who do you trust to talk with about your health?
    a. What makes you trust them?
5. Can you think of a recent conversation you had about your health with someone in your life? Can you describe the conversation?
    a. What made you trust this person to talk with them about your health?

Symptomatic Phase: *[Now we are going to transition to talking about your TB experience. We would like to talk about when you started to feel sick. Can you close your eyes and try to remember when you first started to feel sick?]*

6. When you first started to feel sick, what were your symptoms?
    a. What symptom(s) did you notice the most?
        i. Probe: Why was this symptom more noticeable that the others?
    b. What symptoms did other people notice the most?
        i. Probe: How did this make you feel?
    c. Can you tell me (a story) about how people treated you after they noticed these symptoms?
    d. How did you expect people to treat you after they noticed your symptoms?

    e. Who, if anyone, did you speak with about feeling sick/your symptoms?
        i. Probe: Is this the same person that you spoke about earlier (question 3)?
        ii. What about this person made you trust them to talk about you feeling sick?
        iii. How long after feeling sick did you start talking to them? (i.e when you first coughed or after weeks of coughing?)
        iv. What did you talk about?
            1. Was tuberculosis mentioned specifically? Or a different illness?
        v. How did you feel talking to them?
        vi. What type of support did they provide? (Mental or tangible)
        vii. How did you feel about them providing you support?
    f. If you didn't speak to anyone about feeling sick, did anyone come to speak to you about looking/acting sick?
        i. Who spoke to you?
            1. What is your relationship to them?
        ii. When did they speak to you?
            1. Had you already noticed your symptoms/admit to yourself that you were feeling sick?
        iii. What did they talk to you about?
            1. Was tuberculosis mentioned specifically? Or a different illness?
        iv. How did you feel when they spoke to you?
7. How did your symptoms affect your **daily activities** [referring to symptoms before diagnosis]?
    a. How did your symptoms impact your activities at home?
        i. Probe: Can you provide an example?
        ii. Probe: How were the symptoms influencing your relationships at home?
    b. How did your symptoms impact your activities at work?
        i. Probe: Can you provide an example?
    c. How were your symptoms affecting your social activities?
        i. Probe: Can you provide an example?
    d. How did your symptoms affect your interactions with men in your life? Example
    e. How did your symptoms affect your interactions with women in your life? Example

Seeking Care Phase: *[Now we would like to talk to you about when you were seeking care for your symptoms. Can you close your eyes and try to remember when you first went to the clinic?]*

8. What made you decide to go to the clinic for your symptoms?
    a. What did you think you were sick with?
    b. Before you went to the clinic, what were your thoughts about going to the clinic?
        i. How did you feel about going to the clinic?
            1. What influenced these feelings? (i.e. testing results, seeing friends/neighbours, losing job/income due to clinic visit/diagnosis)
    c. Where else did you consider seeking care for your symptoms other than the clinic? (Explore traditional healers, Spaza shops, pharmacies, other clinics/clinical services)
        i. If they respond affirmatively:
            1. What was it about (__this other place__) that you considered going there?

            2. Did you go to (__this other place__) before or after you went to the clinic?
                a. What made you decide to seek care at [item mentioned above] before going to the clinic?
                b. What did they give you?
        ii. Did you try your own traditional remedies?
            1. (IF YES) Tell me about these traditional remedies?

9. Which clinic did you decide go to go?
    a. What was it about this clinic that you decided to go there?
    b. Had you been to this clinic before?
        i. Probe: For what reason?
    c. Is this the closest clinic to where you stay?
        i. If no, what is it about the clinic closest to where you stay that you decided not to go there?
    d. Is this the closest clinic to where you work?
    e. [FOR MEN] What do you think other **men** in your community think about this clinic?
        i. Do you think other men go to this clinic?
        ii. Do you think they feel comfortable going there?
            1. Probe: Why do you think so?
            2. Probe: What makes clinics uncomfortable?
        iii. How does this effect your decision to go to this clinic?
        iv. Do men talk to each other about which clinic they go to? Please provide an example.
    f. [FOR WOMEN] How do other **women** in your community think about this clinic?
        i. Do other women go to this clinic?
        ii. Do you think they feel comfortable going there?
            1. Probe: Why do you think so?
            2. Probe: What makes clinics uncomfortable?
        iii. How does this effect your decision to go to this clinic?
        iv. Do women talk to each other about which clinic they go to? Please provide an example.
10. What were your experiences with finding time to go to the clinic?
    a. Did you have to take time away from responsibilities to get to the clinic? Please provide an example.
        i. Probe: Work/Jobs?
        ii. Probe: Care taking?
    b. Were there any delays from when you wanted to go to the clinic to when you actually went to the clinic?
        i. If yes/positive response: Can you please explain why there was a delay?
    c. Did anyone accompany you to the clinic?
        i. If yes, who?
        ii. What is it about this person that you trust them to attend the clinic with you?

TB Testing Phase: *[Now I would like to talk to you about when you got tested for TB. I would be grateful if you could walk me through your TB testing experience in the clinic. When thinking about my questions, please include your interactions with nurses, doctors and other clinic staff people]*

11. How many times did you go to the clinic before you were tested for TB?
    a. If more than once, what do you think were the reasons it took this many times?
    b. What or who motivated you to continue to go to the clinic?
12. Think back to the visit when you got tested, as a [man/woman] how did you feel about sitting in the waiting room?
    a. Can you remember and describe who you sat next to?
    b. Did you feel judged while sitting in the waiting room?
        i. Who did you feel was judging you? (other patients, clinic staff?)
    c. What were you thinking about while you were sitting there?
    d. What were you feeling while sitting in the waiting room? Fear? Anxiety? Boredom? Joy?
13. Can you remember and describe when you went into the consultant room? Who did you meet with? *[use this response for the following question]*
14. As a [man/woman] how did you feel when you sat down with the [healthcare provider – use response from above]?
    a. Did the [sister] ask you how you were doing? How did they great you?
    b. Tell me about your interaction with the provider?
    c. How did you explain to the [sister] why you came to the clinic?
    d. Did the sister mention anything to you about TB during this consultation?
15. When the nurse asked you to cough into the bottle, what did they tell you about why they were asking you to do this?
    a. Probe: Did they tell you that this was for TB testing? Please explain.
    b. Probe: Did the [sister] coach you on how to produce a sputum sample?
        i. When did she coach you? Before or only if you were having difficulties producing?
16. Where did you go to cough?
    a. PROBE: [If at clinic] How did you feel having to cough in front of other people at the clinic?
        i. What was your experience with coughing? Please explain how it went.
    b. PROBE: [If at home or away from the clinic] How did you feel having to cough at home?
        i. Why did you decide to take the bottle home?
        ii. What was your experience with coughing [for sputum]? Please explain how it went.
        iii. When did you return to the clinic with your bottle?
17. [FOR WOMEN] Compared to your experience, do you think men would have a similar or different experience?
18. [FOR MEN] Compared to your experience, do you think women would have a similar or different experience?
19. When did the nurse tell you to return for your results?
20. Was there anything about your testing experience- with the clinic or the nurse -that you wished could have changed?

a. What would you advise other [men/women] to do to make sure that they have a positive testing experience?
b. What would you advise the clinic to do to make sure that clients have a positive testing experience?

Waiting Period between Testing and Receiving Results: *[Now we would like to talk to you about when you were waiting for your testing results.]*

21. When you left the clinic after you were tested for TB, how did you feel?
    a. Did you talk to anyone about your experience at the clinic?
        i. If yes, who did you speak with?
            1. What was it about this person that made you trust telling them about your experience at the clinic?
            2. Can you tell me more about your conversation:
                a. What did you tell them?
                b. What did they say to you?
                    a. How did what they say make you feel?
            3. What type of support or advice did they provide? (Mental or tangible)

                If Support/Advice Provided:
                a. Was this support or advice helpful?
                b. How did you feel about them supporting you?

                If Support/Advice Was NOT Provided:
                a. What type of support or advice do you wish they provided you?

        ii. If they did not speak with someone else:
            1. Did you feel that you had to keep your attending clinic a secret?
            2. What is it about your experiences that you felt you had to keep this a secret?
            3. What type of support did you wish someone would have provided you at this time?

Diagnosis and Treatment initiation Phase: *[Now we would like to talk to you about when you were diagnosed with TB and when you first started taking treatment. Can you close your eyes and try to remember this time?]*

22. What or who motivated you to return to the clinic to receive your results?
    a. Did you receive your results within a few days or weeks based on what you remember?
    b. What was it like to go back to the clinic to receive your results?
23. Please think back to the visit when you received your results. As a [man/woman] how did you feel about sitting in the waiting room?
    a. Can you remember and describe what you were feeling while you were sitting in the waiting room?
        i. What were you thinking about while you were sitting there?

b. Did you feel judged while sitting in the waiting room?
    i. Who did you feel was judging you? (other patients, clinic staff?)
    ii. Why do you think they were judging you?

24. Can you please describe your interactions with the nurse when they told you that you had TB?
    a. How did you feel when the sister told you that you had TB?
    b. When the [sister/nurse] told you that you had TB, did they say it with care (empathy or sympathy)?
        i. Probe: Did you feel that you were treated with respect when you received your test results?
    c. Can you tell me about any advice or stories the sister share with you about what it's like to have TB?
        i. What did the nurse mention about disclosing your results to family members or close friends?
25. Was there anything about your experience with receiving your test results that you didn't like or that you wished would have been different?
26. If you could advise the clinic on the best way to tell people that they have tested positive for TB, what would your advice be?
27. After the nurse told you that you had TB, what did they say about when you had to start treatment?
    a. Was there anything specific that you were immediately worried or concerned about?
    b. What were the first thoughts that you had about having to drink pills every day?
    c. What did the nurse advice you about how to take your treatment?
    d. What did the nurse tell you about when and how you collect your pills? (pill pick up and/or in clinic DOTS)
28. Did you start drinking your pills the very same day you got your TB test results?
29. When you left the clinic after you were diagnosed with TB, how did you feel?
    a. How did you feel walking home with your TB pills?
    b. [If NO to 28] Did you talk to anyone before you started your treatment?
        i. If yes, what was it about this person that you trusted to speak with them about starting TB treatment?
        ii. If no, is there anyone that you would have liked to talk to?
30. Who was the first person you spoke with about your TB?
    a. When did you tell this person?
    b. What was it about this person that you trusted to speak with them about your TB?
    c. How did they respond?
    d. How did their response make you feel?
    e. What type of support or advice did they provide? (Mental or tangible)

        If Support/Advice Provided:
        a. Was this support or advice helpful?
        b. How did you feel about them supporting you?

        If Support/Advice Was NOT Provided:
        a. What type of support or advice do you wish they provided you?

31. (If they have children) Did you tell your family and children about your TB?
    a. If yes, how did they react?
        i. How did that make you feel?
    b. If no, what is it about your family that you did not want to tell them about your TB?
32. Did you talk to any men in your life about your TB?
    a. If yes, what was it about these men that made you trust telling them about your TB?
    b. If no, what stopped you from discussing this with the men in your life?
33. Did you talk to any women in your life about your TB?
    a. If yes, what was it about these women that made you trust telling them about your TB?
    b. If no, what stopped you from discussing this with the women in your life?
34. (If employed) Did you tell your employer?
    a. If yes, what was it about your employer that made you trust telling them about your TB status?
    b. What was their reaction?
    c. How did that make you feel?
    d. If no, what stopped you from discussing this with your employer?
35. Did you try to keep your diagnosis a secret at any point?
    a. If yes, who did you try and keep it a secret from?
        i. What was it about these people that you wanted to keep your TB a secret from them?
    b. If no, what made you not want to keep your TB status a secret?

Intensive Treatment Phase *[Now we would like to talk to you about the time after your diagnosis and when you were taking your medication. Can you close your eyes and try to remember this time?]*

36. Can you describe for me how you were feeling mentally and emotionally when you first started taking your treatment?
    a. What type of positive and negative experiences did you have when you first started taking your treatment?
37. [May have already been mentioned] How soon after you started treatment did you tell someone that you **started** treatment for your TB?
    a. Was there anyone that knew about your TB that you didn't tell or that you didn't want to know?
38. Can you tell me about someone in your life that provided you support during this time?
    a. What type of support did they provide?
    b. How was this support helpful to you?
    c. How did it make you feel to receive support from this person?
    d. Who else do you wish had provided you support during this time?
    e. Were there other types of help or support that you wish you would have received?
39. Can you describe how your life changed when you first started taking your treatment?
    a. How did things change in your household after you started treatment?
        i. Did you move in with family? **If yes**:
            1. How did this make you feel?
            2. How did your family feel about you moving in with them?

ii. [If the participant was already living with family] How did your family feel about you staying/living in the home?
iii. Can you describe how your household dynamics changed when you started treatment? (i.e. sleeping arrangements; eating arrangements, etc…)?
1. How did you feel about these changes?
b. Can you remember and describe how your work/employment changed after you started treatment? [If no change, then skip to (c)]
i. Were you able to continue to work?
ii. Did you have difficulties actioning the work you needed to do?
iii. How did you feel about these changes?
iv. Did you ever feel unwelcomed by your co-workers or supervisor because of your TB?
c. What type of financial challenges did you face due to your TB?
i. Did your household experience financial challenges due to your TB?
ii. IF YES: When did you start to experience financial challenges?
iii. IF YES: What could have been done to help you deal with these challenges?
d. Did you ever feel unwelcomed by anyone because of your TB [work place, social place, home]?
i. What could have been done to make you feel welcomed/supported?

40. Can you tell me about your experiences of picking up your medication refills?
a. Were there things that made it more difficult to pick up your medication on time?
b. Were there things that made it easier or less difficult to pick up your medication on time?
c. If we could have made medication pickups easier for you or other people with TB, what would you advise us to do or change?

41. Were you ever visited by a community health worker while you were taking TB treatment?
a. **If yes**, what did they tell you was the reason for their visit?
i. Was there anything about their visit that you wish they had done differently?
ii. Was their anything about their visit that you appreciated?
iii. If we could make these visits more helpful for you or other people with TB, what would you advise or recommend us to do or change?
b. **If no**, do you wish a community health worker would have visited you?
i. What would be part of a helpful visit from a community health worker?
ii. Is there anything about a visit from a community health worker that would NOT be helpful to you?

Continuation phase (Based on treatment stage)

42. Thinking back to when you started to feel better from your TB illness……
a. How long after you started drinking your pills did you start to feel better?
b. What did you notice most about how you were feeling?
i. When you started to feel better, how did this make you feel?
ii. How was it different taking your pills when you started feeling better?
iii. Did your feeling better effect how you took your medication?

43. When did other people start to notice that you were feeling/doing better?

44. Please help me remember if you had relationships that changed because of your TB
    NOTE: List all relationships that they mention.
    a. Can you give me an example of a person with whom your relationship changed when they found out you had TB, and then changed back to “normal” (i.e., when you we not sick with TB)?
    b. How did your relationships change with other [men/women/family] in your life?
45. Can you tell me about someone in your life that provided you support during this time?
    a. What type of support did they provide?
    b. How was this support helpful to you?
    c. How did it make you feel to receive support from this person?
    d. Who else do you wish had provided you support during this time?
    e. Were there other types of help or support that you wish you would have received?
46. Can you tell me about when you felt that your life was returning to normal?
    a. When did you go back to work?
    b. When did you start to socialize with other people again (family/friends)?
47. Based on your experiences, what would you advise other people to do or not do after they started to feel better?

Continue with different questions by participant type, below:

Recently Defaulted	***Recently Completed***	***Currently on Treatment***
We noticed that there was a period in which you did not go back to the clinic to pickup your TB pills.<br><br>1. Can you tell me what happened during this period that you were not drinking your pills? (WAIT FOR ANSWER)<br><br>2. Was there something happening in your life that impacted your ability to pick up your medication at the clinic? (WAIT FOR ANSWER)<br>a. Probe: What was the biggest factor that impacted your ability to continue drinking your pills?<br>b. What type of support do you wish you would have had at this time to help you remain in care and drinking your pills?	1. Can you tell me about things in your life that may have made it difficult for you to always take your treatment?<br>a. Are there things that you did not have that made it difficult to always take your medication?<br>b. Are there things you wish you had that would have helped to make it easier to always take your medication?<br><br>2. Are there people in your life that helped you stay committed to completing your full treatment?<br>a. What type of support did they provide that helped you so much?<br>b. Did you find it difficult to always accept support from these people?<br><br>3. Can you tell me about a time when you just wanted to stop drinking your pills?	1. Can you tell me about things in your life that may make it difficult for you to always take your treatment?<br>a. Are there things that you do not have that make it difficult to always take your medication?<br>b. Are there things you wish you had that would help to make it easier to always take your medication?<br><br>2. Are there people in your life that help you stay committed to taking your treatment every day?<br>a. What type of support do they provide that helps you so much?<br>b. Do you find it difficult to always accept support from these people?<br><br>3. Can you tell me about a time when you wanted to stop drinking your pills?<br>a. PROBE: What made this time so difficult for you to want to keep going with your TB treatment?<br>b. What kept you motivated to keep drinking your pills?

3. Did you tell anyone about your challenges with continuing to drink your pills?

   IF YES:
   a. What was it about this person that you trusted to tell them about your challenges?
   b. What did they say when you told them that you stopped drinking your pills?
   c. Did they provide you any type of support at this time?
   d. Did this support help you decide to start drinking your pills again?
   e. What type of support do you wish this person would have provided you to help you through this period, remain in TB care, and continue drinking your pills?

   IF NO:
   a. Was there anyone you wish you could have spoken to during this time?
   b. What is it about this person that you wish you would have spoken with them?
   c. What type of support do you wish you would have received from anyone to help you through this period, remain in TB care, and continue drinking your pills?
   d. Thinking back, do you think there was anything you could have done differently or wish you would have

   a. PROBE: What made this time so difficult for you to want to keep going with your TB treatment?
   b. What kept you motivated to keep drinking your pills?
   c. Was there someone that you confided in about wanting to stop drinking your pills?
   d. What was it about this person that you trusted to tell them about wanting to stop drinking your pills?
   e. What did you talk with them about?
   f. What type of support did you have from family or friends that helped you stay committed to completing your treatment?

4. What where the biggest motivators for you to continue and finish your TB treatment?
   a. How did you remind yourself of these motivators?
   b. Did anyone help remind you?
   c. Where there people that made it difficult to stay motivated?
   d. PROBE: What was it about these people that made you demotivated?

5. What were the most important resources that you had that helped you to remain engaged in care and to complete your course of treatment?

6. What advice would you give to other men/women who are struggling to stay in care and keep drinking their pills?

We know that many people struggle to remain in care and consistently drink their TB pills. As someone who successfully completed their entire course of TB treatment, we want your advice.

   c. Was there someone that you confided in about wanting to stop drinking your pills?
   d. What was it about this person that you trusted to tell them about wanting to stop drinking your pills?
   e. What did you talk with them about?
   f. What type of support did you have from family or friends that helped you stay committed to taking your treatment?

4. What are the biggest motivators for you to continue your TB treatment?
   a. How do you remind yourself of these motivators?
   b. Does anyone help remind you?
   c. Are there people that make it difficult to stay motivated?
   d. PROBE: What is it about these people that demotivate you?

5. What are the most important resources you have to help you remain engaged in care and keep drinking your pills?

6. What advice would you give to other men/women who are struggling to stay in care and keep drinking their pills?

We know that many people struggle to remain in care and consistently drink their TB pills. As someone who has remained engaged in TB care and treatment, we want your advice.

7. If we were to develop a special support program for other men/women to remain in TB care and complete their treatment:
   a. What would you include in the support program?
   b. What would you NOT include in the program?
   c. How would you deliver this program to them?
   d. Who would be the people that would delivered this program?
   e. Where would they deliver it?

known that would have helped you stay in TB care and keep drinking your pills?<br>e. What type of additional support would have helped you stay engaged in your treatment during this time?<br><br>IF THE SAY ANYTHING ABOUT STOPPING MEDICATION BECAUSE THEY WERE FEELING BETTER, ASK:<br><br>4. You mentioned that you stopped drinking you pills, in part, because you were feeling better. Had you started to feel sick again, what would you have done?<br><br>5. If we were to develop a special support program for other men/women to remain in TB care and complete their treatment:<br>a. What would you include in the support program?<br>b. What would you NOT include in the program?<br>c. How would you deliver this program to them?<br>d. Who would be the people that would delivered this program?<br>e. Where would they deliver it?	7. If we were to develop a special support program for other men/women to remain in TB care and complete their treatment:<br>a. What would you include in the support program?<br>b. What would you NOT include in the program?<br>c. How would you deliver this program to them?<br>d. Who would be the people that would delivered this program?<br>e. Where would they deliver it?	

Concluding Remarks

Thank you for your time and for your willingness to discuss your experience with us. We appreciate all that you have shared today.

Cheat Sheet for Additional Probes: Use these probes throughout the interview. We expect participants may bring these issues up but more probing will be needed to generate their discussion.

- If mention work: How did TB impact your work? Provide an example. Do you think work would be good place to do a TB project with men?
- If mention communicating on mobile phone about TB: Do you enjoy talking/comfortable talking about TB over--- or SMSing on mobile phones about TB testing and treatment (something like this but less clinical). Provide an example. Would you be interested in a TB project that used mobile phones?
- If mention anything about 'being strong' or manhood related: How does TB impact being strong? Provide an example.
- If mention resources (food, money, housing), ask how these impact their TB care.
- If mention taverns or shebeens: Do you talk about TB there? Provide an example. Do you think TB project for men would work in said tavern or shebeen?
- If mention attending church: Do you think that church is a good place to do a TB project for men? Why?
- If mention household or community roles changing or concern of change: How did you think about your role in your household after you learned that you had TB? Or, how did TB treatment impact your role in your household?
