# Supplementary material for: Social isolation, social exclusion, and access to mental and tangible resources: mapping the gendered impact of tuberculosis-related stigma among men and women living with tuberculosis in Eastern Cape Province, South Africa
Source: BMC Glob Public Health. 2025 Jun 5;3:50. doi: 10.1186/s44263-025-00166-6 (PMC12142910; doi:10.1186/s44263-025-00166-6)
Supplement: Supplementary file 4 — Additional file 4. Final Codebook. [file 44263_2025_166_MOESM4_ESM.pdf]

Id	Parent Id	Depth	Title	Description	Applications
1		0	Alternative Delivery of Treatment		90
2	1	1	Community (Com)	Expresses desire to have TB treatment done at location other than clinic or at home	7
3	1	1	Home Delivery (HD)	Expresses desire to have TB treatment delivered at home	78
4		0	Association with HIV	Any mention of HIV	89
5		0	Barriers to Access/Treatment		31
6	5	1	Holidays (H)	Participants describe that the holiday season was the reason they forgot to take pills/could not comply with the treatment	2
7	5	1	School/Work (S/W)	Participants describe school work/employment as a reason for not complying with required TB treatment	16
8		0	Change in Behavior'-' Self (CBS)		125
9	8	1	CBS Neg	When s/he changes their behavior negatively in response to TB illness	27
10	8	1	CBS Pos	When s/he changes their behavior positively in response to TB illness	81
11		0	Change in Behavior'-'Others (CBO)		111
12	11	1	CBO Neg	When others change their behavior negatively in response to participant's TB illness	47
13	11	1	CBO Pos	When others change their behavior positively in response to participant's TB illness	50
14		0	Clinic Expectations		67
15	14	1	Negative expectations	Refers to when participants explain what they fear the clinic will be like	25
16	14	1	Positive expectations	Refers to when participants explain what they hope the clinic will be like	41
17		0	Clinic Experiences		676
18	17	1	Clinic capacity (CC)	Refers to when s/he mentions something lacking/something that could be improved re: clinic's capacity (capacity includes cleanliness and service provision)	62
19	17	1	CLINIC Pill pickup (PP)	Refers to how s/he was told to pick up pills from the clinic	98
20	17	1	Hours of Operation '+'/'-' (HO)	Refers to having high access to one's clinic of choice	0
21	17	1	Privacy Y/N (P)	Refers to whether the participant reported having adequate privacy at clinics	37
22	17	1	Repeat Testing Y/N (RT)	Refers to whether the particpant reported having /needing multiple TB tests prior to diagnosis being made	56
23	17	1	Reputation (REP)	Refers to when s/he mentions something lacking/something that could be improved re: the clinic's reputation	131
24	17	1	Sputum production CLINIC/HOME (SP)	Refers to whether sputum collection was done at the clinic or at home	151
25	17	1	Wait time (WT)	Reported experiences with wait times at clinic	129
26	17	1	Waiting room '+'/'-' (WR)	Reported experience at the waiting room of the clinic	103
27		0	Clinic Preferences		241
28	27	1	Clinic type'-'Day Clinic (CT'-'C)	Prefers to visit day clinic type setting for care	40
29	27	1	Clinic type'-'Hospital (CT'-'H)	Prefers to visit hospital type setting for care	35

30	27	1	Goes for other things (OTH)	Mentions going to clinic for non'-'TB related issues	100
31	27	1	Location'-'Far (L'-'F)	Prefers clinic to be far from residence	19
32	27	1	Location'-'Nearby (L'-'N)	Prefers clinic to be near residence	126
33		0	Clinic Provider Communication		183
34	33	1	Empathy (E)	Participant reports having an interaction with a healthcare provider where the provider expressed empathy and listened	177
35	33	1	No Empathy (NE)	Participant reports having an interaction with a healthcare provider where the provider did not listen and was unfriendly.	9
36		0	Community Awareness Meetings	S/he suggests holding community meetings to provide TB education	38
37		0	COVID'-'19	Participant mentions COVID'-'19/anything related to the pandemic or lockdowns. Please include codes for isolation etc. if participant mentions it.	75
38		0	Disclosure Skill Building Received (DSBR)		69
39	38	1	DSBR No	Refers to when participants did NOT receive skills to help disclose TB status to others	44
40	38	1	DSBR Yes	Refers to whether participant received skills to help disclose TB status to others	22
41		0	Discriminatory Actions		120
42	41	1	Anticipated Change (AC)	When the participant anticipates/expects a change in others' behaviors towards them as a result of TB status	32
43	41	1	Familial Ostracization (FO)	When the participant's family behaves in a way that excludes the participant from activities they were not excluded from prior to TB diagnosis	9
44	41	1	Gossip (G)	Participant reports experiencing/hearing about others gossip about him/her due to TB status	45
45	41	1	Job loss (JL)	When the participant reports losing their job as a result of being diagnosed with TB	5
46	41	1	Loss of housing (LH)	Participant reports losing housing/experiences a change in housing status as a result of TB diagnosis/treatment	0
47	41	1	Social Ostracization (SO)	When the participant's social group behaves in a way that excludes the participant from activities they were not excluded from prior to TB diagnosis	28
48	41	1	Teasing (T)	When participant describes being teased or mocked because of their TB diagnosis/treatment status	19
49		0	Emotional State Changes		530
50	49	1	Aggressive (A)	Refers to being mad or angry all the time.	18
51	49	1	Guilty (G)	Refers to guilt about transmitting TB to others.	5
52	49	1	Happy (H)	Refers to being happy or normal.	220
53	49	1	Less Energy (LE)	S/he reports having less energy over the course of the TB treatment	68
54	49	1	Relieved (RE)	Refers to feeling good about changes or decision.	106

55	49	1	Shame (S)	Refers to feeling bad about being TB positive.	34
56	49	1	Strong (ST)	Refers to feeling strong mentally and can make decisions.	116
57	49	1	Weak (W)	Refers to feeling mentally unwell or unable to make decisions.	30
58		0	Employment	Employment/Unemployment status	120
59	58	1	Employed	Mentions being formally employed (DO NOT use when odd jobs employment is mentioned)	19
60	58	1	Unemployment'-'No Job	Mentions being unemployed/not having a job/being a student and not having a job	80
61	58	1	Unemployment'-'Odd Jobs	Mentions being employed to do odd jobs (DO NOT use when formal employment is mentioned)	30
62		0	Family & Living Environment	Components of living environment	259
63	62	1	Cleanliness/Hygiene	Mentions things that contribute to the living environment being clean or hygenic	64
64	62	1	Conflict	Mentions that he has conflicts or instabilities with friends and family.	17
65	62	1	Dirtiness/Mess		59
66	62	1	Living Alone	Mentions that he/she lives alone in primary household	17
67	62	1	Living in communal compound or with friends	Mentions that he/she lives in a communal compound or group setting (etc. compounds for laborers or hostel for students) or with friends.	1
68	62	1	Living with parents/family	Mentions that s/he lives with mother, father or siblings	86
69	62	1	Living with Partner	Mentions that s/he is living with partner with or without children	23
70		0	Judgement'-'others (JO)		105
71	70	1	JO'-'Neg	When others makes a negative judgement about the participant	58
72	70	1	JO'-'Pos	When others makes a positive judgement about the participant	33
73		0	Judgement'-'Self (JS)		148
74	73	1	JS judgement '-' NEG	When s/he makes a negative judgement about themselves	25
75	73	1	JS judgement '+' POS	When s/he makes a positive judgement about themselves	120
76		0	Key Supporter Access & Frequency		43
77	76	1	High A&F	Refers to KS being highly accessible'-'-'always or almost always available, high frequency of interaction between KS and the participant	40
78	76	1	Low A&F	Refers to KS being less accessible'-'-'not always available, low frequency of interaction between KS and the participant	3
79		0	Key Supporter Behaviors		798
80	79	1	Caring (C)	Refers to whether s/he feels cared for by KS or not	235
81	79	1	Clinic/check'-'up reminders (CUR)	Refers to whether the KS reminds her/him to go to the clinic when needed	66
82	79	1	Confiding (CO)	Refers to whether s/he can confide in the KS about TB related issues	130
83	79	1	Joking (J)	Refers to whether s/he can joke around with KS	5
84	79	1	Medication reminders (MR)	Refers to whether KS reminds her/him to take their TB medication as necessary	160

85	79	1	Motivation (MO)	Refers to whether motivation is provided to participant from KS re: TB care or not	451
86		0	Key Supporter Changes	Refers to whether the KS changes from one person to a different person over the course of the participant's TB care	32
87		0	Key Supporter Modality		27
88	87	1	Modality'-' In Person (M'-'IP)	Refers to whether s/he does or does not receive supportive in'-'person interactions from others	9
89	87	1	Modality'-'Phone Calls (M'-'PC)	Refers to whether s/he does or does not receive supportive calls from others	16
90	87	1	Modality'-'Texts (M'-'T)	Refers to whether s/he does or does not receive supportive texts from others	2
91		0	Monetary Tangible Resources	Tangible Resources related to having/needing money	177
92	91	1	Having Fares	When participant mentions HAVING money/fares to transport themselves to their clinic visits for their TB treatment	8
93	91	1	Housing Resources		8
94	93	2	Housing '+'/'-'	Mentions either stable housing (code as '+') or transitionary housing (moving from place to place, no regular primary household'-'code as '-')	3
95	93	2	Space '+'/'-'	Mentions having or not having adequate personal space within one's residence	3
96	91	1	Money'-'Individual	When participant mentions having or not having their own money for TB related expenses	39
97	91	1	Money'-'Social	When participant mentions needing or using monetary resources that comes from someone else (not the participant)	75
98	91	1	Needing Fares	When participant mentions NEEDING fares to transport themselves to their clinic visits for their TB treatment (mentions needing money for this)	53
99	91	1	Rides	When participant mentions NEEDING a ride to the clinic for their TB treatment from someone else with a car	18
100		0	Peer to peer	S/he suggests having peers who completed TB treatment support others	29
101		0	Person Type Support		694
102	101	1	Aunt (A)	When s/he indicates their KS is their aunt	57
103	101	1	Boyfriend (Bo)		76
104	101	1	Brother (B)	When s/he indicates their KS is their brother	30
105	101	1	Child (C)	When s/he indicates their KS is their child	33
106	101	1	Community Health Worker (CHW)	When s/he indicates that their preferred supporter is a community health worker rather than someone they know personally	14
107	101	1	Cousin (CO)	When s/he indicates their KS is their cousin	29
108	101	1	Father (F)	When s/he indicates their KS is their father	17
109	101	1	Female friend (FF)	When s/he indicates their KS is their female friend	53
110	101	1	Girlfriend (Gr)		82
111	101	1	Grandfather (GF)	When s/he indicates their KS is their grandfather	1

112	101	1	Grandmother (GM)	When s/he indicates their KS is their grandmother	18
113	101	1	Male friend (MF)	When s/he indicates their KS is their male friend	48
114	101	1	Mother (M)	When s/he indicates their KS is their mother	165
115	101	1	Neighbor (N)		26
116	101	1	Sister (S)	When s/he indicates their KS is their sister	142
117	101	1	Uncle (U)	When s/he indicates their KS is their uncle	13
118		0	Physical Health Resources		424
119	118	1	Cooking '+'/'-' (CO)	Mentions being able or unable to cook food for self/mentions having or not having someone else to cook food for them	87
120	118	1	Food Security '+'/'-' (FS)	Mentions having or not having adequate food on a daily basis	100
121	118	1	Medication '+'/'-' (M)	Mentions being able or unable to access/acquire any and all medication, including but not limited to TB medication	12
122	118	1	Physical Strength/Weakness '+'/'-' (PS)	Mentions being having or not having enough strenght to walk oneself to the TB clinic when needed	62
123	118	1	Sleep '+'/'-' (S)	Mentions getting or not getting adequate sleep regularly	41
124	118	1	Smart Phone Access '+'/'-' (SP)	Mentions having or not having access to smart phones vs. regular phones vs. no phone	1
125	118	1	Weight Change '+'/'-' (WC)	Participant reports changes in their weight throughout the course of their TB treatment	165
126		0	Physical State Changes		274
127	126	1	Less Strength (LS)	S/he reports having less strength over the course of the TB treatment	40
128	126	1	More Energy (ME)	S/he reports having more energy over the course of the TB treatment	25
129	126	1	More Strength (MS)	S/he reports having more strength over the course of the TB treatment	41
130	126	1	Timing of change (T)	S/he reports when s/he started seeing a change in symptoms (ie. 2 weeks after starting medication or 3 weeks etc.)	156
131		0	Self'-'Support		331
132	131	1	Coping Skills for Stigma (CSS)	Refers to needs to navigate stigma related experiences (TB and HIV). Related to gossip experience or perceived	42
133	131	1	Family Protection (FM)	Mentions that s/he is motivated to seek TB care/treatment to protect family members from TB	40
134	131	1	Resilience (RES)	Mentions being positive/continuing care'-'seeking; depending on one's self to be motivated for treatment completion	262
135		0	Stop smoking or drinking	S/he suggests support to stop smoking or drinking	111
136		0	Support Evaluation		105
137	136	1	High Support	Participant felt that support from KS was adequate throught their TB treatment	94
138	136	1	Key Supporter Trust		237

139	138	2	KS High Trust	Refers to participant having a high degree of trust in the KS re: their TB care	234
140	138	2	KS Low Trust	Refers to participant having a low degree of trust in the KS re: their TB care	3
141	136	1	Low Support	Participant felt that support from KS was not enough throught their TB treatment	4
142		0	TB Disclosure		463
143	142	1	Disclosure to aunt (A)	When participant discloses or desires to disclose TB status to aunt	23
144	142	1	Disclosure to brother (B)	When participant discloses or desires to disclose TB status to brother	36
145	142	1	Disclosure to cousin (C)	When participant discloses or desires to disclose TB status to a cousin	12
146	142	1	Disclosure to Employer		32
147	142	1	Disclosure to Family (Fam)		121
148	142	1	Disclosure to father (F)	When participant discloses or desires to disclose TB status to father	7
149	142	1	Disclosure to grandfather (GF)	When participant discloses or desires to disclose TB status to grandfather	2
150	142	1	Disclosure to grandmother (GM)	When participant discloses or desires to disclose TB status to grandmother	6
151	142	1	Disclosure to husband/boyfriend (H)	When participant discloses or desires to disclose TB status to husband/boyfriend/male partner	44
152	142	1	Disclosure to mother (M)	When participant discloses or desires to disclose TB status to mother	55
153	142	1	Disclosure to Neighbor or Friend (N)		72
154	142	1	Disclosure to No One (NO)	When S/he says that they didn't or won't disclose their TB status to anyone.	5
155	142	1	Disclosure to sister (S)	When participant discloses or desires to disclose TB status to sister	68
156	142	1	Disclosure to uncle (U)	When participant discloses or desires to disclose TB status to uncle	8
157	142	1	Disclosure to wife/girlfriend (W)	When participant discloses or desires to disclose TB status to wife/girlfried/female partner	41
158		0	TB education and Disclosure		206
159	158	1	No TBED	Refers to when they did not receive education about TB at the clinic PLUS DISCLOSURE SKILLS	53
160	158	1	TB Treatment Illness Process Knowledge (TTIPK)	Refers to mentions of TB treatment expectations, example: when someone gets ill by taking treatment at the start and then feels better at month 2 or 3.	40
161	158	1	Yes TBED	Refers to when they received education about TB at the clinic PLUS DISCLOSURE SKILLS	112
162		0	TB Illness Discussion		14
163	162	1	TBI'-'Bad (B)	Refers to DESIRE OR regular conversations with Key Supporters to talk about personal symptoms and health changes negatively	1
164	162	1	TBI'-'Good (G)	Refers to DESIRE OR regular conversations with Key Supporters to talk about personal symptoms and health changes positively	11
165		0	TB Social Impact		285
166	165	1	Impact on education (ED)	How TB impacts participant's education	9
167	165	1	Impact on exercise (EX)	How TB influences exercise	2

168	165	1	Impact on lifestyle (L)	How TB impacts participant's lifestyle (ie. Sleep, diet etc.)	104
169	165	1	Impact on relationships with men (RM)	How men percieve participant's illness	42
170	165	1	Impact on relationships with women (RW)	How women percieve participant's illness	45
171	165	1	Impact on work (W)	How TB impacts participant's employment or unemployment	113
172		0	TB Symptoms		555
173	172	1	Changes in symptoms (CS)	S/he reports how symptoms changed over time (ie. Better, worse, stayed the same)	151
174	172	1	Chief complaint (CC)	S/he describes their main, most urgent symptom	126
175	172	1	Reported symptoms (RS)	S/he reports all symptoms they believed to be associated with TB	215
176	172	1	Symptom severity (SS)	S/he reports how badly symptoms affected them	170
177		0	TB'-'Emotions'-'Community		32
178	177	1	TB Anger'-'Community	When someone in the community expresses anger about TB diagnosis/treatment	4
179	177	1	TB Fear'-'Community	When someone in the community expresses fear about TB diagnosis/treatment	16
180	177	1	TB Isolation'-'Community	When someone in the community expresses that TB treatment has caused isolation	13
181	177	1	TB Worry'-'Community	When someone in the community expresses worry about TB diagnosis/treatment	2
182		0	TB'-'Emotions'-'Family		31
183	182	1	TB Anger'-'Family	When someone in the family expresses anger about TB diagnosis/treatment	3
184	182	1	TB Fear'-'Family	When someone in the family expresses fear about TB diagnosis/treatment	11
185	182	1	TB Isolation'-'Family	When someone in the family expresses that TB treatment has caused isolation	4
186	182	1	TB Worry'-'Family	When someone in the family expresses worry about TB diagnosis/treatment	14
187		0	TB'-'Emotions'-'Self		217
188	187	1	TB Anger'-'Self	When s/he expresses anger about TB diagnosis/treatment	14
189	187	1	TB Fear of Judgement'-'Self	When s/he expresses fear of judgement about TB status	33
190	187	1	TB Fear'-'Self	When s/he expresses fear about TB diagnosis/treatment	65
191	187	1	TB Isolation'-'Self	When s/he expresses that TB treatment has caused isolation	49
192	187	1	TB Worry'-'Self	When s/he expresses worry about TB diagnosis/treatment	62
193		0	TB'-'Specific Support		46
194	193	1	Educated Support Network (ESN)	Refers to having different people in social network providing support based on their TB knowledge.	18
195	193	1	TB Knowledge Lead (TKL)	Refers to social network leaders or others serving as TB experts to inform and support TB testing and treatment.	26
196		0	Websites Interactive	S/he suggests creating a TB education website where one can link to services too	3
